# An insight in to microwave induced defects and its impact on nonlinear process in NiO nanostructures under femtosecond and continuous wave laser excitation

**DOI:** 10.1039/d4ra06056c

**Published:** 2024-09-20

**Authors:** Ramseena Thundiyil, P. Poornesh, K. Ozga, J. Jedryka

**Affiliations:** a Department of Physics, Manipal Institute of Technology, Manipal Academy of Higher Education Manipal Karnataka 576104 India poornesh.p@manipal.edu poorneshp@gmail.com; b Faculty of Electrical Engineering, Czestochowa University of Technology Armii Krajowej 17 PL-42-201 Czestochowa Poland

## Abstract

This work demonstrates the impact of microwave (MW) irradiation on third-order nonlinear optical (NLO) processes in chemically deposited NiO nanostructure films. The optical nonlinearity of the NiO nanostructure films was studied using third-harmonic generation (THG) measurements in the pulsed femtosecond laser regime and the Z-scan technique in the continuous wave laser regime. In the ultrafast pulsed regime, THG measurements revealed a significant increase in the THG signal of MW-irradiated NiO nanostructures due to photoexcitation and relaxation processes, resulting from an enhancement in defect concentration. This increase in defect density upon MW irradiation was quantified by PL and XPS studies. Under continuous wave laser irradiation, the Z-scan technique showed an enhanced absorption coefficient of ∼10^−1^ m W^−1^ and a nonlinear refractive index of ∼10^−7^ m^2^ W^−1^. The high NLO values in both pulsed and continuous laser regimes indicate that MW-irradiated NiO nanostructure films hold promise for optoelectronic device applications.

## Abstract 2

This work demonstrates the impact of microwave (MW) irradiation on third-order nonlinear optical (NLO) processes in chemically deposited NiO nanostructure films.

## Graphical abstract

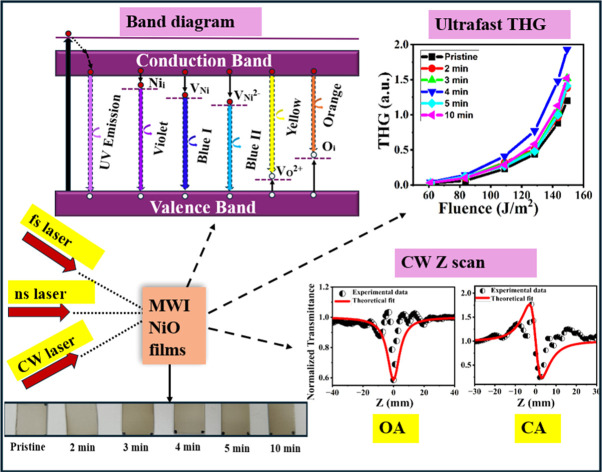

## 1. Introduction

Nanostructure films are pivotal in the realm of photonics due to their extensive range of optical applications. The nanoscale dimensions of these films significantly enhance their optoelectronic properties due to unique physical and optical characteristics at this scale. Nanomaterials exhibit size-dependent properties, including increased surface-to-volume ratios, quantum confinement effects, and enhanced light–matter interactions, thereby improving electron–photon interactions.^1,2^ These characteristics lead to improved performance in various promising fields, such as photonics, optoelectronics, telecommunications, biomedical optics, and nonlinear optics (NLO).

In the field of NLO, researchers have extensively studied the effects of high-intensity light on nanomaterials.^3–5^ These studies have revealed the exceptional capabilities of nanomaterials for multiphoton absorption and high nonlinear absorption coefficients. These properties contribute to strong and swift responses in NLO materials, making them ideal for advanced applications. The nonlinear optical properties of metal oxides and doped metal oxide thin film nanoparticles have been rigorously investigated using various spectroscopic techniques.^6–8^ The unique NLO properties observed in these materials are being harnessed for applications in optical limiting, optoelectronics, telecommunications, and optical switching devices.^9–11^ NiO semiconductor stands out among various transparent metal oxides due to its wide bandgap and electronic, optical, and magnetic properties, making it valuable for interdisciplinary applications. Known for its high stability, both chemically and physically, as well as its low toxicity, and durability, NiO has a significant impact in various fields. In optoelectronics, NiO is recognized for its role in solar cells,^12^ photodetectors,^13^ and light-emitting diodes.^14^ When subjected to intense, coherent laser beams, NiO nanostructure films exhibit remarkable nonlinear optical properties.^9,15^

Post-deposition treatment techniques such as annealing and irradiation have been extensively studied for their ability to enhance the properties of NiO materials. High-energy beams with high-frequency irradiation, such as electron beams, gamma rays, and ultraviolet rays, are powerful tools for modifying the microstructural properties of materials. These beams have been extensively explored in various applications, particularly in the fields of electronics, sensing, and optoelectronics.^16,17^ They offer several advantages, such as the capacity to conveniently adjust the optical and structural properties of materials. Electron beams, gamma rays, and ultraviolet rays can induce significant changes in the properties of materials, making them highly desirable for a diverse array of applications. In addition to their utility in modifying the properties of materials, high-energy beams also offer several practical advantages. For instance, they can be precisely controlled to achieve the desired level of modification, allowing for fine-tuning of the material's properties. Numerous studies have reported that irradiation modifies the physical, chemical, and optical properties of NiO nanostructure films, leading to enhanced optical responses. In view of the mentioned distinctiveness of NiO films, many researchers have been attempting to explore their structural, optical, and NLO properties. M. Rashad *et al.*^17^ synthesized NiO nanoparticles *via* microwave irradiation and observed that UV exposure significantly reduces the optical bandgap by creating defects and disorders. They also noted that the nanoparticles exhibited enhanced optical nonlinearities associated with quantum size effects. P. Mallick *et al.*^18^ prepared NiO thin films on Si substrates using the electron vaporization method and subjected them to swift ion irradiation. The authors observed that swift ion irradiation improved the crystallization and texturing of the films at intermediate fluences, with changes in the crystallinity and texturing of NiO grains depending on the initial microstructure of the film. A. Qasem *et al.*^19^ investigated nanostructured NiO thin films treated with laser pulses ranging from 0 to 150, with intervals of 50 pulses. The energy band gaps were found to be lowered and the band tail energies to be extended upon exposure of laser light. SEM micrographs showed holes and cracks due to the laser breaking bonds between Ni^2+^ and O^2−^ ions. The calculations confirmed the optical bandgap contraction and tail energy expansion with increasing pulses, attributed to increased defects. K. Jouini *et al.*^20^ prepared NiO thin films using spray pyrolysis and subjected them to gamma irradiation ranging from 180 Gy to 10 kGy. Structural studies revealed a phase transition from NiO to Ni_2_O_3_ beyond 5 kGy. At a 10 kGy dose, the films exhibited a combination of NiO and Ni_2_O_3_ phases, with optical studies showing activity in both the UV and visible ranges. This combination enhanced the photocatalytic performance under gamma ray treatment. Recent research has demonstrated that various irradiation methods can significantly modify the structural, optical, morphological, and nonlinear optical properties of NiO thin films. The findings from these studies confirm that irradiation enhances the material's properties.

Furthermore, these beams can be utilized across a diverse array of materials, establishing them as versatile tools in materials science and engineering. But sometimes, these high energy beams are found to destroy the materials also. This is where microwave (MW) irradiation, a low-frequency irradiation, offers a less destructive and more cost-effective approach to material modification. MW irradiation has emerged as a powerful tool in various fields due to its unique advantages over conventional heating methods.^21^ It offers several advantages over other forms of irradiation, such as rapid and uniform heating, higher yields, and reduced energy consumption.^22^ These benefits have led to its widespread adoption in research and industrial applications. Here, in this study, we are going to explore the new novelty field of the influence of MW irradiation on NiO nanostructure films. In this study, we need to emphasize the method that we employed to prepare for nanostructure NiO films. We utilized the air-assisted chemical spray pyrolysis method to prepare NiO nanostructured films. This method is renowned for its ability to produce uniform films and is favored for its simplicity and cost-effectiveness. Several deposition parameters include the flow rate of the spray, the amount of solution sprayed onto the substrate, the substrate temperature, and the distance between the nozzle and the substrate significantly influence the quality and uniformity of the nanostructure films. Optimizing these parameters is crucial for producing films, which attributes distinguish it from other deposition methods, making it a highly attractive option for the deposition of NiO nanostructure films.

The literature on third-order nonlinear optical effects in NiO nanostructures is limited. Additionally, comparative studies on the influence of NiO nanostructure films under continuous wave and pulsed laser regimes are not well-established. To address this gap, our recent study focused on synthesizing NiO nanostructure films using spray pyrolysis, followed by an extensive examination of their NLO response in NiO nanostructures using both CW and femtosecond pulsed lasers. Importantly, we aim to understand the NLO processes in systems treated with microwave irradiation and examine the impact of MW irradiation on both CW and pulsed laser regimes. Therefore, this research investigates the impact of microwave irradiation on the nonlinearity and thermo-optic properties of NiO nanostructure films, utilizing both pulsed and continuous wave laser regimes.

## 2. Experimental details

### 2.1. Materials used

Microscopic glass slides were acquired from Labtech. Acetone, 2-propanol (EMPLURA grade), and nickel acetate tetrahydrate (98% purity) were sourced from MERCK. Deionized water (18.3 MΩ) was used throughout all procedures. All chemical compounds were utilized to prepare the precursor solution for spray pyrolysis technique.

### 2.2. Nanostructure thin film deposition

Microscopic glass slide substrates were cleaned sequentially with soapy water, deionized water, acetone, and isopropanol (IPA) for 10 minutes each using an ultrasonicate. Subsequently, the substrates were dried under a nitrogen flow. To ensure the removal of organic contaminants and reduce surface roughness, the substrates were then subjected to ozone treatment for 10 minutes, resulting in a cleaner and more uniform surface.

The aqueous precursor solution was prepared by dissolving 1.2442 g of nickel acetate tetrahydrate (Ni(CH_3_COO)_2_·4H_2_O) in 100 mL of deionized water, followed by stirring for one hour to ensure a homogeneous and clear solution. This solution was then loaded into the spray pyrolysis syringe for direct use in NiO deposition. The substrate temperature was maintained at a constant 450 °C during the deposition process. The spray pyrolysis software controlled the spray head's motion along both the *x* and *y* axes. The solution was sprayed onto the glass substrate placed on the substrate holder, where it underwent pyrolysis, forming a thin film. The spraying nozzle and the substrate were spaced 19 cm apart with a solution being sprayed flow rate of 2 mL min^−1^. Air served as the carrier gas at 1 mbar. With 2 mm intervals, the ultrasonic nozzle moved in an S-shaped pattern in the *x* and *y* axes, maintaining a steady speed of 30 mm s^−1^. During deposition, the temperature varied by ±10 °C around 450 °C.

The nickel acetate tetrahydrate precursor solution decomposed and pyrolyzed at 450 °C, resulting in solid nickel oxide, while carbon dioxide and water were released as gases.

1 Ni(CH_3_COO)_2_·4H_2_O → NiO + CO_2_↑ + H_2_O↑

The resulting film is light brown in color. The thickness of the film was ascertained through the utilization of a stylus profilometer, the film thickness was determined to be ∼250 nm.

### 2.3. Microwave irradiation

**Fig. 1:**
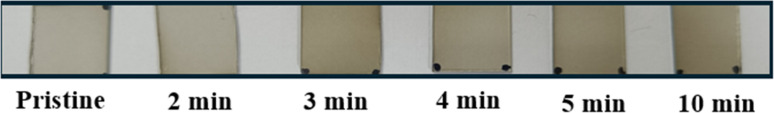
NiO nanostructure films at various MW irradiations.

**Fig. 2:**
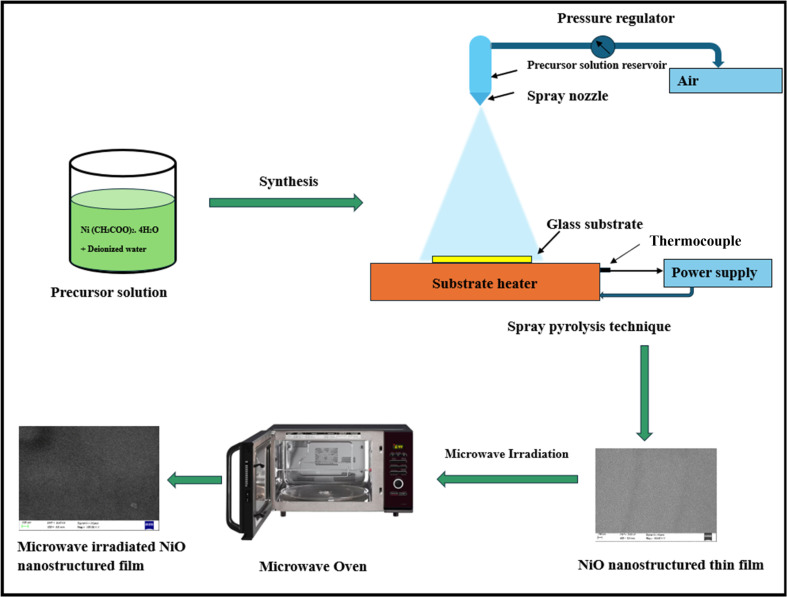
Schematic representation of process flow for preparation of microwave irradiated NiO nanostructure films.

The NiO nanostructure films were subjected to microwave irradiation using a commercially available microwave oven (LG, model number: MC2146BRT) operating at a frequency of 2.45 GHz. The samples were positioned at the center of the microwave chamber on a rotating table. The microwave power was fixed at 800 W for irradiation. The irradiation time varied between 2, 3, 4, 5, and 10 minutes respectively. After each irradiation, the sample was allowed to cool, and it was observed that the sample became darker grey as the irradiation increased. Fig. 1 illustrates the changes observed in NiO nanostructure films upon MW irradiation. The schematic representation of process flow for preparation of microwave irradiated NiO nanostructure films, depicted in Fig. 2.

### 2.4. Characterization of nanostructure films

#### 2.4.1. Structural characterization

The deposited films' crystal structure was studied using a Rigaku SmartLab diffractometer with Cu Kα radiation with 40 kV and 30 mA, scanning from 30° to 90° at a rate of 1° min^−1^. The deposited films underwent analysis for their composition, phase, impurity levels, and defects using LabRAM HR (UV) Raman spectroscopy with a 514 nm laser and CCD detector at room temperature.

X-ray photoelectron spectroscopy (XPS) was utilized for the assessment of the oxidation state of the elements in the deposited films. The analysis was performed using an AXIS ULTRA system with a monochromatic Al Kα X-ray excitation source operating at 14 kV. The C 1s signal at 284.8 eV was used as the reference binding energy.

#### 2.4.2. Morphological and optical characterization

Both Field Emission Scanning Electron Microscopy (FESEM) and Atomic Force Microscopy (AFM) were employed to study the morphology and surface features of the deposited thin films. FESEM analysis was conducted using a ZEISS ULTRA55 scanning electron microscope with a dispersive energy X-ray (EDX) analyzer, operated at 20 kilovolts, after sputtering a thin layer of gold onto the samples. AFM observations were made using an Innova SPM atomic force microscope in tapping mode.

The nanostructure deposited films were analyzed for UV-visible light transmission using a 1900i UV-vis spectrophotometer, ranging from 190 to 1100 nm, with glass substrates providing the background. Photoluminescence studies were conducted to detect defects in the films, using a JASCO FP-8300 spectrofluorometer. Measurements were taken from 320 nm to 750 nm at ambient temperature, with an excitation wavelength of 300 nm, acquiring an emission spectrum for the thin films.

#### 2.4.3. Third-order nonlinear optical processes

**Fig. 3:**
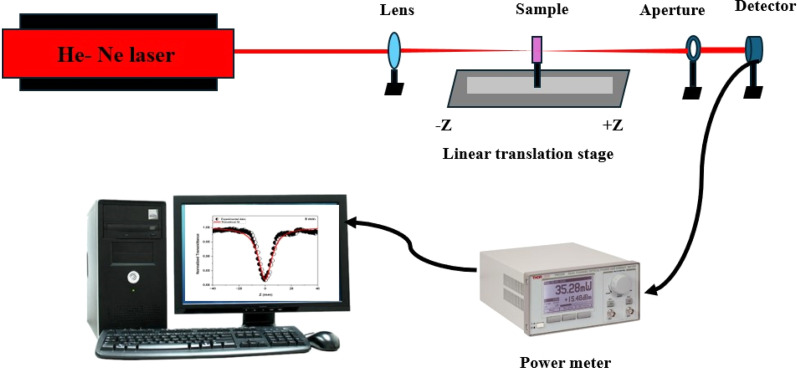
Schematic representation of CW Z scan experimental setup.

To explore the nonlinear optical properties of the deposited film, we conducted NLO experiments using Z-scan techniques. When a high-intensity laser passes through a material, it exhibits nonlinear behavior concerning the electric field. To study the effects of films when subjected to laser light, we conducted a Z-scan experiment.^23^Fig. 3 depicts the configuration of the Z-scan experiment. The experimental setup of the Z-scan technique includes a laser, convex lens, translating moving sample holder, aperture, and a detector. The experiment involves two methods to determine the nonlinear absorption (NLA) and nonlinear refraction (NLR) of the material. The open-aperture Z-scan method provides information on NLA, while the closed-aperture method gives the NLR of the material. In this experiment, we used a continuous wave He–Ne laser known for its high stability and low noise. Operating at a fixed single wavelength of 632.8 nm, it offers a consistent and well-characterized light source for nonlinear optical studies. The laser light passes through the convex lens and then the sample-translating stage. A convex lens with a focal length of 5 cm is used to focus the laser beam onto the sample. The sample was moved from −5 cm to +5 cm in both *z*-directions around the focal point of the convex lens using a linear translation stage. The laser light that passes through the sample is detected by the detectors. These detectors are crucial for accurately measuring the transmitted laser power. They are connected to a power meter (THOR LABS PM320E), which in turn is connected to a PC for data recording and analysis. The movement of the linear translator is controlled by the Z-scan software. In the open-aperture (OA) configuration, the laser beam is directed through a convex lens focus it onto the sample. The detector measures the entire transmission through the sample. In the closed-aperture (CA) configuration, the beam that has passed through the sample is directed through an aperture positioned ahead of the detector. This aperture is placed to ensure that any nonlinear phase shift experienced by the beam is solely due to the sample. In the current study, the normalized transmittance value *S* = 0.7.

In this report, the NiO nanostructure film sample is irradiated by an intense continuous wave He–Ne laser with an input intensity of 20 mW. During the experiment, the beam is passed through a 5 cm convex lens, which focuses the beam on the sample. The obtained beam waist at the focal point and Rayleigh length were 35.11 μm and 6.11 mm, respectively. The thin film thickness meets the sample requirement *L* ≪ *Z*_R_, in which the Rayleigh range (*Z*_R_). The sample thickness is less than the Rayleigh length *Z*_R,_ hence the thin film approximation is valid. Then, Z-scan experiments were conducted on the plane glass substrate, revealing no significant nonlinear contribution at the applied input power levels. Consequently, the glass substrate's impact on the observed nonlinearity was deemed negligible. A linear translation stage was employed to scan the NiO thin film in both positive and negative *z*-directions surrounding the convex lens's focal point. The scan range was ±5 cm. The laser power transmitted through the NiO film is utilized to measure both the nonlinear refractive index *n*_2_ and the nonlinear absorption coefficient *β*, with and without aperture, in front of the detector, accordingly. The detector is connected to power meters, utilized for measuring the laser power that passes through.

#### 2.4.4. Third harmonic generation

**Fig. 4:**
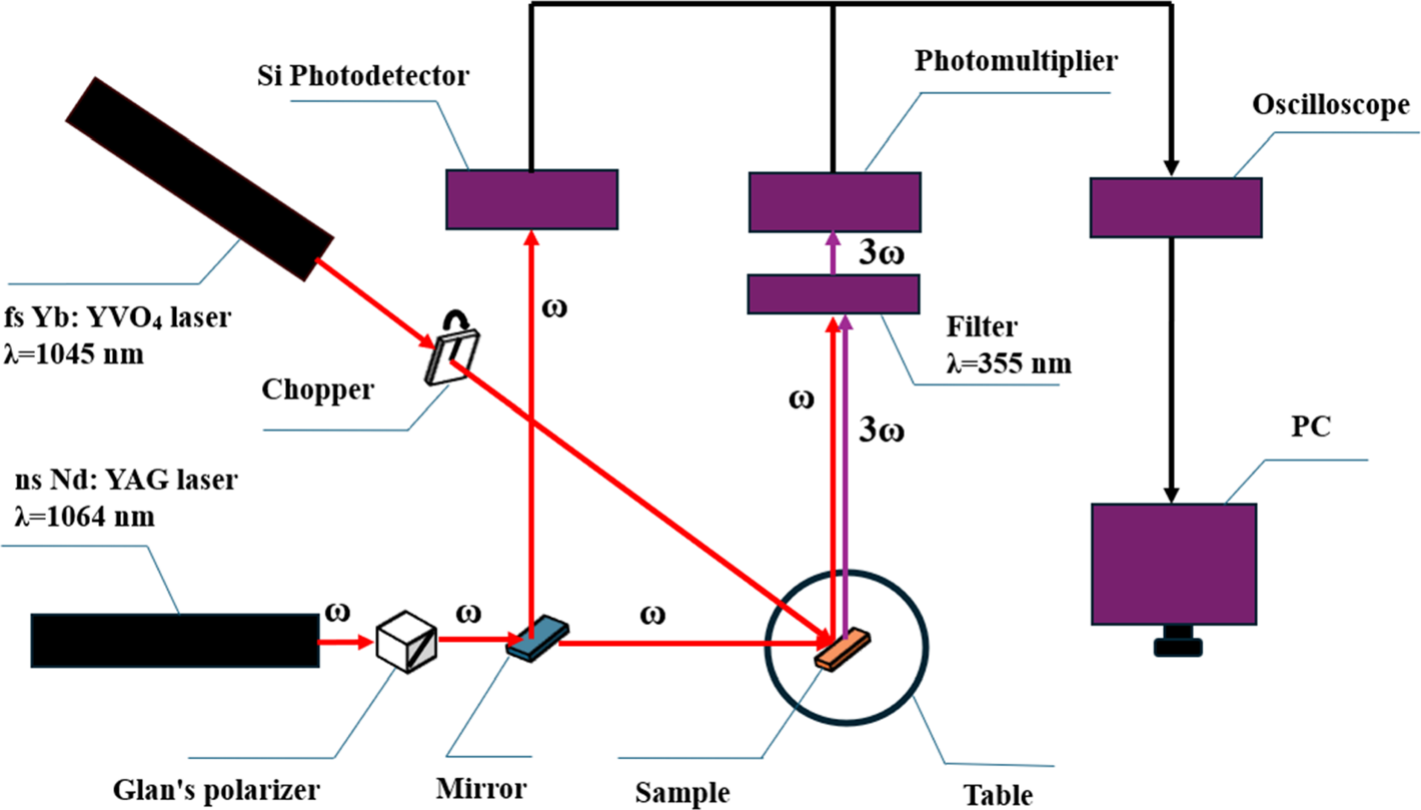
Experimental setup for laser-induced THG measurement.

The third harmonic signal of the light beam from deposited nanostructured NiO thin films was measured using the third harmonic generation (THG) technique. Third harmonic signals are generated when a fundamental frequency beam passes through a nonlinear material, producing a frequency that is three times that of the input. To investigate the third harmonic response of the material, we conducted measurements using the THG method, as depicted in Fig. 4. The experimental setup comprises two pulsed lasers: a femtosecond Yb:YVO_4_ laser with a wavelength of 1045 nm and a nanosecond Nd:YAG laser with 1064 nm wavelength. The setup also includes a chopper, Glan's polarizer, mirrors, a silicon photodetector, a rotating sample holder, an interference filter for 355 nm wavelength, a photomultiplier tube, an oscilloscope, and a PC for data acquisition and analysis.

In this experiment, to determine the third harmonic signal of the material, we employed a femtosecond (fs) laser as the pumping laser. The fs laser used was a Yb:YVO_4_ laser with a wavelength of 1045 nm, a repetition frequency of 63 MHz, and a maximum energy of 40 nJ. This fs laser induces changes in the dipole moment of the sample, thereby altering the electronic states of the material. The high intensity and ultrashort pulses of the fs laser generate high peak powers necessary for strong nonlinear interactions. To prevent thermal damage to the samples due to prolonged exposure to the fs laser, a chopper was placed in the path of the laser beam. The chopper, with a single slit 2 mm wide, rotates at a speed of 1 revolution per second, effectively modulating the pulsed laser beam that falls on the sample. A nanosecond (ns) laser was also utilized, with its spot size corrected to approximately 1 mm to match that of the femtosecond laser, ensuring that both beams interact with the same area of the sample. This uniformity is crucial for consistent and reliable third harmonic generation (THG) measurements. The ns laser served as the probing laser beam, detecting changes induced by the pumping beam. The probing beam used was an Nd:YAG laser with a wavelength of 1064 nm, an 8 ns pulse duration, and a repetition frequency of 10 Hz. The laser beam had a diameter of 8 mm and a maximum energy of 150 J m^−2^.

The laser beam then passed through a Glan's polarizer placed in front of the beam path. The Glan polarizer ensured that the probing beam (from the ns laser) was highly polarized and allowed modulation of the light beam's intensity reaching the sample, leading to the tuning of the output fundamental energy density. The polarizer was capable of withstanding laser damage at 4 GW cm^−2^. We adjusted the polarizer angle in 5 degree increments to control the 1064 nm laser's energy reaching the NiO nanostructure film. This adjustment allowed us to detect the THG signal at the lowest possible energy density of the ns laser.

Following, the light passed through a transmissive mirror that reflected a portion of the incident light while transmitting the rest. This mirror directed part of the ns laser beam to a silicon photodetector (Si PD) and allowed another part to pass through to the sample surface. The reflective portion of the mirror directed a segment of the probing beam to the Si PD, used to synchronize the measurement system. The transmissive portion of the mirror allowed the probing beam to reach the sample surface, where it interacted with the changes in optical dipole moments induced by the fs pumping laser. The resulting THG signal then passed through an interference filter with a wavelength of 355 nm, which exclusively allowed the THG signal to pass through.

The THG signal was then recorded by a photomultiplier tube followed by an oscilloscope. The Tektronix MSO 3054 oscilloscope, with a high sampling rate of 2.5 GS s^−1^, provided high-resolution measurements of the fast-changing signals from the laser interactions, capturing both the fundamental signal (1064 nm) and the THG signal. The oscilloscope was connected to a PC for data acquisition and analysis. The whole experimental system was bounded in a box to eliminate the impact of external unwanted light dispersion, ensuring that the THG measurements reflected the true interactions within the sample, providing reliable and high-quality data.

## 3. Results and discussions

### 3.1. XRD analysis of MW irradiated NiO nanostructure films

**Fig. 5:**
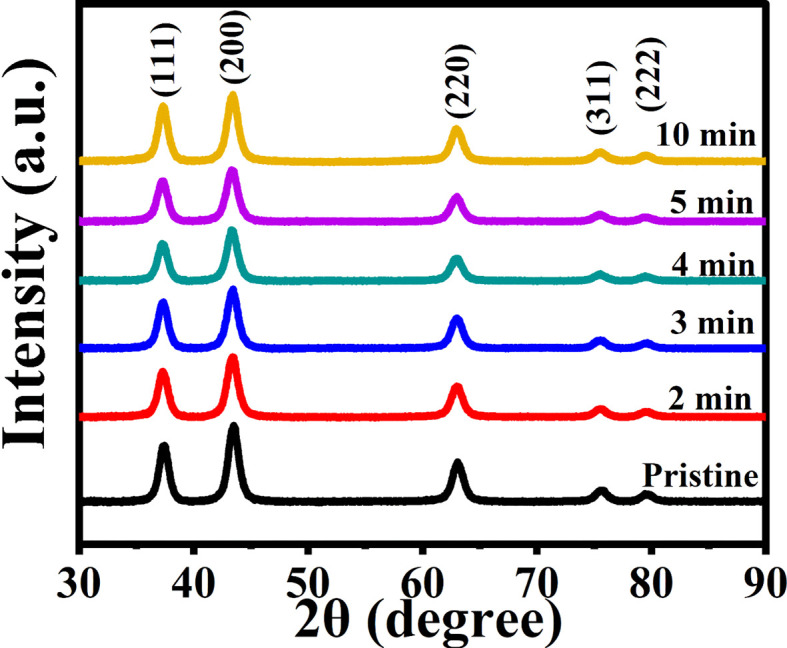
The diffraction spectra of pristine and MW irradiated NiO nanostructure films.

To investigate the crystal structure and phase of the prepared nanostructured films, structural studies were conducted using X-ray diffraction (XRD) analysis. The XRD patterns were obtained for both pristine and microwave irradiated NiO nanostructure films, as depicted in Fig. 5. The diffractograms reveal the presence of multiple peaks, indicating the polycrystalline nature of the films. All observed peaks correspond to the face-centered cubic structure of NiO, as indexed with the standard JCPDS file number 01-073-1519. The diffraction spectrum features three prominent peaks corresponding to the (111), (200), and (220) planes, along with two smaller peaks at the (311) and (222) planes. The polycrystallinity is evident in all samples. The preferential peak is towards the (200) plane, which is the most intense peak. Increasing the MW irradiation led to noticeable alterations in both peak intensity and full width at half-maximum (FWHM). Specifically, the intensity decreased with 5 minutes of MW irradiation but increased again for the 10 minute irradiated sample. These variations are attributed to the MW heating effect, which influences the orientation and arrangement of the lattice planes. Furthermore, the smaller plane peaks diminished with increased MW irradiation. The broadening of FWHM suggests an increase in strain within the film due to the MW heating effect. The observations, such as changes in intensity and FWHM, validate the structural alterations occurring in NiO thin films during MW exposure. These variations in intensity and FWHM may be attributed to the increased defect concentration resulting from dislocation or distortion within the film induced by irradiation.^24^

A minimal peak shift (∼0.13°) towards lower angles was observed in samples irradiated for 2 to 10 minutes compared to the pristine samples, implying thermal expansion in the lattice parameters caused by MW heating. Furthermore, this alteration in the diffraction peak position is associated with changes in the local atomic environment of the Ni^2+^ ions within the crystal lattice. Such changes can be attributed to structural modifications, such as lattice distortion, strain,^25^ or the introduction of defects induced by MW irradiation. No additional peaks, which could indicate secondary phases or impurities, were detected after MW irradiation, confirming the purity of the samples. These findings demonstrate that MW irradiation affects the lattice atoms orientations and strain within the NiO thin films without introducing impurity phases.

The structural properties of the prepared nanostructured films, specifically the crystallite size and microstrain, were determined using two prominent analytical methods: the Scherrer method and the size–strain plot (SSP) method.

#### 3.1.1. Scherrer method

The Scherrer method estimates the crystallite size within the material based on the peak broadening observed in the X-ray diffraction (XRD) pattern, primarily attributing this broadening to the finite size of the crystallites. The crystallite size (*D*) is calculated using the Scherrer equation:2
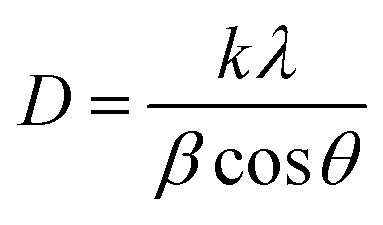
where *λ* is the wavelength of the XRD radiation, *β* is the full width at half maximum (FWHM) of the peak, *θ* is the Bragg angle, *k* is the shape factor, typically close to 0.9 for spherical particles.

#### 3.1.2. Size–strain plot

The SSP method enables the simultaneous calculation of crystallite size and micro strain, assuming that peak broadening results from both crystallite size and strain effects. In this method, the size-induced broadening is described by a Lorentzian profile, while the strain-induced broadening follows a Gaussian profile. The SSP method utilizes the following equation:3
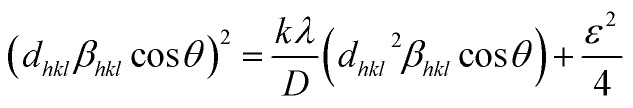
where *d*_*hkl*_ is the interplanar spacing, *β*_*hkl*_ is the FWHM of the peak corresponding to the (*hkl*) planes, *ε* represents the microstrain.

**Fig. 6:**
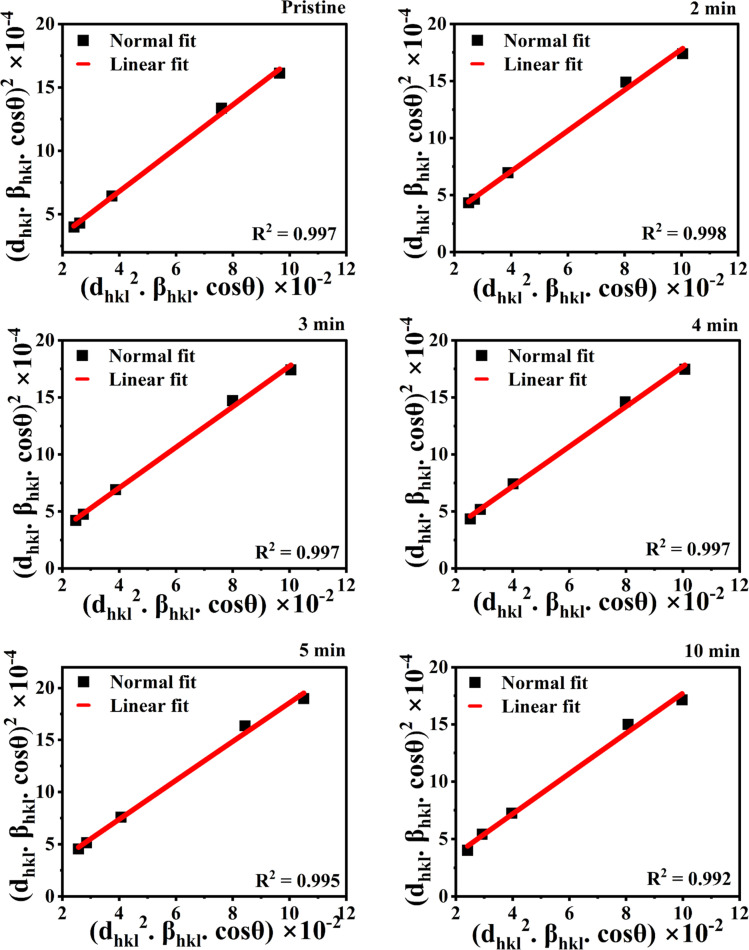
SSP plot of pristine and MW irradiated NiO nanostructure films.

In the size–strain plot, (*d*_*hkl*_*β*_*hkl*_ cos *θ*)^2^ is plotted on the *Y*-axis and (*d*_*hkl*_^2^*β*_*hkl*_ cos *θ*) on the *X*-axis, is depicted in Fig. 6. The slope of the plot provides the crystallite size, while the intercept gives the strain within the films. The SSP method focuses more on reflections at lower angles, where the accuracy and precision of XRD data are typically higher.

The crystallite sizes calculated using both the Scherrer method and the size–strain plot method are nearly equal, and both methods show consistent trends. The SSP method is more reliable and appropriate for analyzing the NiO nanostructure films because it minimizes the influence of less precise data and shows a good linear fit, indicating isotropic variations accurate in the crystallite size and strain of the NiO nanostructures upon MW irradiation. The crystallite size decreases with increasing microwave irradiation, from 8.24 nm to 7.47 nm according to the Scherrer method, and from 8.48 nm to 7.76 nm according to the SSP method. This reduction in crystallite size is attributed to the breaking up of crystallite size due to the MW heating effect. Irradiation can lead to the generation of heat in the material and influence the mobility of atoms in the crystal lattice, affecting the growth and stability of crystallites.^26,27^ Consequently, the irradiation period induces alterations in the film's microstructure by disrupting its original orderly arrangement.^17^

The dislocation density, which is a measure of point defects in the material, represents the number of dislocations per unit length and is calculated using the formula:4
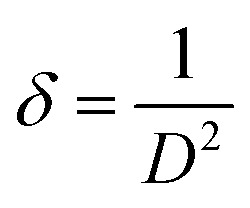


After MW irradiation, the dislocation density of the film was found to increase, indicating that the levels of dislocation, distortion, and defects within the films have been enhanced. This increase is attributed to the MW heating effect, which induces more dislocations in the lattice.

The lattice parameter for the cubic structure is given by:5
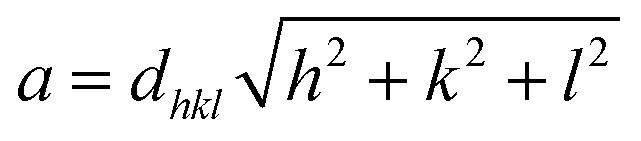
where (*hkl*) are the Miller indices of the planes corresponding to each peak in the XRD patterns. Observations show that there is no significant change in lattice parameters with increased irradiation. Any small changes in the lattice parameter may be due to lattice compression induced by thermal expansion from the MW heating effect.

Strain within the films was calculated using the equation:6
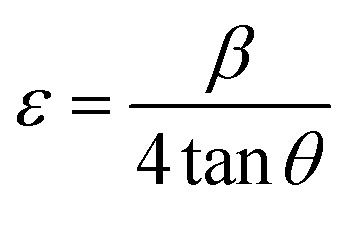


The films exhibit an increase in strain, which is associated with the elevated presence of defects induced by MW irradiation. However, a considerable difference in strain values was observed between the Scherrer and SSP methods. This indicates that the SSP method offers a more comprehensive analysis by considering strain. The number of crystallites per unit volume was determined using:7
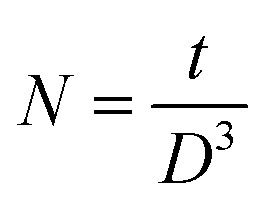
where *t* is the film thickness. An increase in the number of crystallites after MW irradiation suggests that MW irradiation effectively modifies the microstructure of the nanostructure films.

The structural parameters values are depicted in Table 1 and it reveals that MW irradiation has been shown to increase dislocation density, induce strain, and enhance the number of crystallites in the films. These changes reflect significant modifications to the films' microstructure, which can influence their overall physical and chemical behavior.

**Table 1:** Calculated structural parameters of pristine and MW irradiated NiO nanostructure films

MW irradiation	Crystallite size (nm)		Dislocation density (×10^16^ lines per m^2^)		Strain (×10^−2^)		Number of crystallites per unit volume *N* (×10^17^ m^−2^)		Lattice constant *a* (Å)
	Scherrer	SSP	Scherrer	SSP	Scherrer	SSP	Scherrer	SSP	
Pristine	8.24 ± 0.02	8.48 ± 0.02	1.47	1.39	1.19	0.35	4.47	4.09	4.16
2 min	7.82 ± 0.02	8.12 ± 0.02	1.64	1.52	1.25	0.46	5.24	4.67	4.17
3 min	7.86 ± 0.02	8.14 ± 0.02	1.62	1.51	1.25	0.45	5.14	4.63	4.17
4 min	7.90 ± 0.02	8.27 ± 0.02	1.60	1.46	1.24	0.88	5.07	4.43	4.17
5 min	7.47 ± 0.02	7.76 ± 0.02	1.79	1.66	1.31	0.57	6.00	5.34	4.17
10 min	7.79 ± 0.02	8.24 ± 0.02	1.65	1.47	1.26	0.77	5.29	4.47	4.17

### 3.2. Raman analysis of MW irradiated NiO nanostructure films

**Fig. 7:**
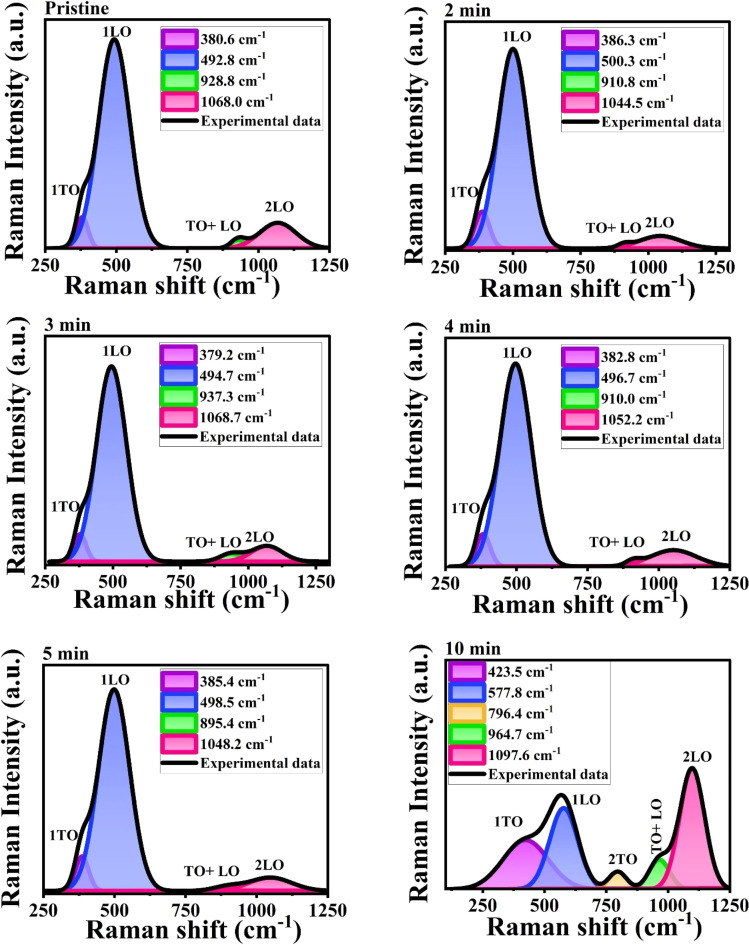
Deconvoluted Raman spectra of MW irradiated NiO nanostructures films.

Raman spectroscopy was employed to investigate the vibrational modes and structural properties and to identify impurities in the films. This technique provides detailed information about the material's crystal structure and different vibrational modes. Fig. 7 illustrates the Gaussian deconvolution of Raman spectra of pristine and MW irradiated NiO nanostructure films, obtained using a 532 nm laser at room temperature over a scanning range from 200 to 1300 cm^−1^. The spectra, plotted as Raman intensity *versus* Raman shift, revealed the presence of five phonon modes characteristic of NiO films. These phonon modes include both first-order phonon modes (1TO and 1LO) and second-order phonon modes (2TO, LO + TO, and 2LO).

#### 3.2.1. First-order Raman phonon modes

The first-order Raman phonon modes, including the Transverse Optical (TO) mode at 380.6 cm^−1^ and the Longitudinal Optical (LO) mode at 492.8 cm^−1^, are observed due to parity-breaking bonds caused by nickel vacancies at the zone boundary. Typically, stoichiometric NiO is inactive for first-order phonon modes. The existence of these modes in the spectra indicates non-stoichiometry in the material, attributed to nickel defects including nickel vacancies,^28^ or the presence of Ni^3+^ ions in the films.^29^ Additionally, they are induced by oxygen interstitials, surface defects, and imperfections occurring in the nanocrystalline films.^30^ The LO bands arise from Ni–O bonding, specifically related to the stretching bonds in the NiO octahedra. Microwave irradiation enhances the intensity and area of the Raman LO mode, indicating its interaction with NiO materials. This irradiation induces a stochastic distribution within the NiO lattice, increasing disorder and enhancing phonon scattering, which broadens the Raman peaks.

The TO phonon modes exhibit a redshift from 380.6 to 423.5 cm^−1^ upon MW irradiation, with the FWHM increasing from 47 to 216 cm^−1^. The area of the TO mode is highest for the 2 minute irradiated sample, suggesting significant lattice distortion or the presence of nickel defect levels, corroborated by XRD spectra. The LO phonon modes shift from 492.8 to 577.8 cm^−1^, with an increased FWHM, attributed to the interaction of MW irradiation with NiO material. Similar observations have been reported by A. Sunny *et al.*^31^ The shifts observed in the TO and LO modes indicate the presence of vacancies and structural defects induced by MW irradiation.^32^ The movement of Raman peaks towards higher wavenumbers suggests an increase in defect concentration within the film after MW irradiation, possibly due to lattice defects introduced by the MW heating effect on the NiO structure.^33^

#### 3.2.2. Second-order Raman phonon modes

The second-order Raman vibration modes, 2TO, TO + LO, and 2LO, result from combinations of first-order bands.^34^ For the pristine sample, the TO + LO and 2LO bands occur at 928.8 and 1068.0 cm^−1^, respectively. The 2LO mode also indicates stretching vibrations of the Ni–O bond in the NiO lattice.^35^ For 10 min irradiated sample, new peaks characteristic of NiO arise at 796.4 cm^−1^, attributed to the 2TO mode. The weak intensity of the 2TO and TO + LO modes suggests that the synthesized materials have a nanocrystalline structure.^36^ Additionally, the intensities and areas of the 2LO mode non-monotonically increase upon MW irradiation, confirming enhanced disorder or defect presence in the films and aligning with earlier XRD results. The dependence of MW irradiation on phonon mode frequencies and FWHMs is summarized in Table 2.

**Table 2:** Summary of Raman peak fitted parameters of MW irradiated NiO nanostructures

MW irradiation	1TO		1LO		LO + TO		2LO	
	FWHM	Area	FWHM	Area	FWHM	Area	FWHM	Area
Pristine	47 ± 1	2038.9 ± 56	130.0 ± 0.4	37 280 ± 92	59 ± 5	687 ± 57	151 ± 61	5328 ± 95
2 min	63.4 ± 0.7	10 772 ± 148	131.6 ± 0.2	120 942 ± 1198	53 ± 6	802 ± 122	182 ± 1 4	10 571 ± 228
3 min	51.2 ± 1	3207 ± 80	130.6 ± 0.3	58 114 ± 112	97 ± 7	1763 ± 169	134 ± 5	4815 ± 187
4 min	57.8 ± 0.8	7341 ± 128	131.1 ± 0.2	104 020 ± 192	56 ± 5	1011 ± 123	191 ± 4	12 285 ± 244
5 min	55.6 ± 0.7	7124 ± 102	130.0 ± 0.2	97 519 ± 159	115 ± 10	1790 ± 257	176 ± 5	8810 ± 293
10 min	216 ± 6	7429 ± 27	134 ± 1	7639 ± 25	97 ± 3	2018 ± 73	118.9 ± 0.9	10 052 ± 78

In the pristine sample to the 5 minute MW-irradiated sample, the one-phonon 1LO mode exhibits higher intensity due to the presence of defects or surface defects. However, as the irradiation is extended to the 10 minute sample, the two-phonon LO band becomes more broadened compared to the 1P mode band. This increased broadening indicates higher disorder and defect levels within the nanostructure film. Additionally, the broad Raman signal supports the presence of oxygen vacancies and nickel-related defects.^37^ A similar behavior of the phonon-related Raman bands in nanosized material was observed in the study by Mironova *et al.*^38^ The shift of all Raman bands to higher wavenumbers indicates increased compressive strain in the films.^39^ Notably, the 10 minute sample shows significant peak shifts due to MW heating, with all phonon modes shifting to higher wavenumbers which is associated with the size-induced phonon confinement, defects, and surface relaxation.^36^ The shift in the 2LO peak from 1068 to 1097.6 cm^−1^ suggests the presence of crystal defects, such as oxygen and nickel vacancies, which can lead to a redshift of the Raman peak. Additionally, the modes broaden with increased MW irradiation due to higher nickel or interstitial oxygen vacancy concentrations, resulting in smaller particle sizes. Raman studies verifies that the prepared nanostructure films have a cubic NiO structure without any impurity or secondary phases, consistent with XRD results. In conclusion, these results imply that MW irradiation induces changes in the defect in the lattice of the material. This highlights the significant impact of MW irradiation on the material's crystallinity and defects.

### 3.3. XPS analysis of MW irradiated NiO nanostructure films

**Fig. 8:**
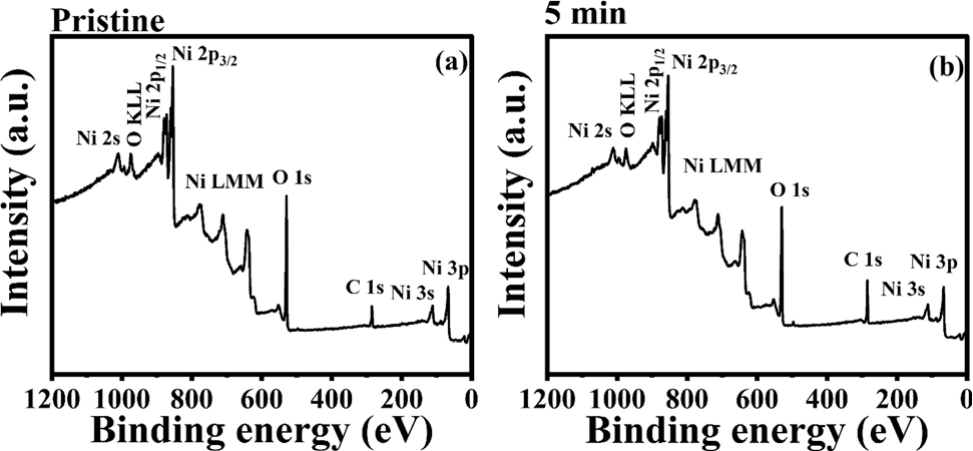
XPS survey spectra of pristine and 5 min MW irradiated NiO nanostructure films.

The chemical analysis of the sample was further carried out to determine the chemical state of the prepared NiO nanostructured NiO films. The XPS studies were conducted to analyze the chemical states and to identify the chemical compositions present in the prepared pristine and MW irradiated NiO nanostructure films. The Fig. 8 represents the XPS survey scan of the pristine and 5 min MW irradiated NiO nanostructure films.

#### 3.3.1. XPS survey

The XPS spectrum revealed photoemission intensity peaks corresponding to Ni 3p, Ni 3s, O 1s, Ni 2p, Auger electron peaks (Ni LMM and O KLL), and the C 1s peak. The binding energies (BE) were as follows: ∼1011 eV for Ni 2s, ∼853 eV for Ni 2p, the ∼(776–640) eV range for Ni LMM peaks, ∼112 eV for Ni 3s, and ∼67 eV for Ni 3p. The order of these peaks reflects the electronic levels of the Ni element, with the inner-shell Ni 2s appearing at a very high BE and decreasing BEs observed as we move to the valence shell electrons.

No foreign elements were detected in the wide spectrum, indicating the grown film has the desired NiO composition, consistent with XRD and Raman analyses. To account for the charging effect in the film on XPS, the binding energies were calibrated using the C 1s peak at 284.8 eV. The core spectra were plotted after correcting for the charging effect using this reference.

#### 3.3.2. Ni 2p core spectra

**Fig. 9:**
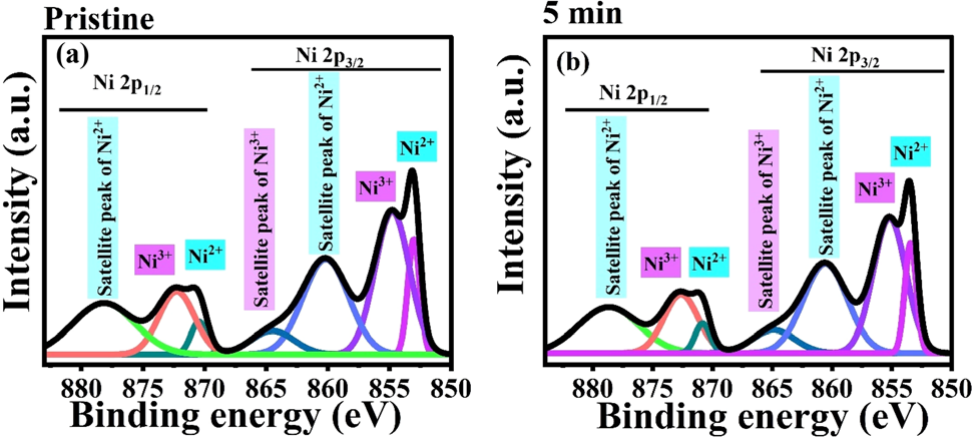
Ni 2p core level spectra of pristine and 5 min MW irradiated NiO nanostructure film.

The Ni 2p core level spectra of Gaussian deconvoluted pristine and 5 min MW irradiated NiO nanostructure films are shown in Fig. 9. The spectra consist of two Ni 2p components: Ni 2p_3/2_ and Ni 2p_1/2_. The peak splitting observed in the spectra is due to spin–orbit coupling. As a result of this coupling, the intensities of the split peaks are in a 2 : 1 ratio, which is clearly visible in the survey spectra. This intensity ratio is determined by the number of electrons in each state, reflecting the degeneracy of the elements.

The binding energy of the Ni 2p_3/2_ component ranges from 850 to 868 eV, while the Ni 2p_1/2_ component ranges from 868 to 883 eV. In the Ni 2p_3/2_ region, four peaks are observed at 853.0, 854.8, 860.2, and 864.6 eV, corresponding to Ni^2+^, Ni^3+^, and the shake-up satellite peaks of Ni^2+^ and Ni^3+^, respectively, in the pristine NiO nanostructure film. The major peak at 853.0 eV and its satellite at 860.2 eV indicate Ni–O bonding in the Ni^2+^ state. The peaks at 854.8 eV and its satellite at 864.5 eV are associated with the Ni^3+^ state, indicating nickel vacancy defects in the film. For the Ni 2p_1/2_ region, binding energies from 868 to 883 eV are observed. Peaks at 870.4, 872.2, and 878.2 eV correspond to Ni^2+^, Ni^3+^, and the satellite peak of Ni^2+^, respectively. The occurrence of Ni^2+^ and Ni^3+^ states signify stoichiometric and non-stoichiometric NiO. Increased Ni 3d–O 2p hybridization and d–d transition mixing are the causes of the satellite peaks in the spectrum.^40^

In the 5 min MW irradiated sample, the Ni 2p_3/2_ binding energy peaks are observed at 854.4, 855.2, 860.6, and 864.9 eV. The Ni 2p_1/2_ binding energy peaks are observed at 870.8, 872.6, and 878.65 eV. After MW irradiation, the core spectra show a shift toward higher binding energies, which is attributed to the formation of defects in the NiO lattice. These defects alter the ionic charge distribution, leading to cation vacancies and changes in the chemical composition, particularly oxygen concentration.^41^ The peak broadening and shifts are also linked to the charging effect, which is induced by microwave irradiation.

The detailed analysis of the decomposed XPS spectrum indicates that the peak intensity for the core levels of Ni^3+^ (2p_3/2_ and 2p_1/2_) is higher than that for Ni^2+^ (2p_3/2_ and 2p_1/2_) in both pristine and 5 minute irradiated samples. The intensity ratio of Ni^3+^ to Ni^2+^ peaks (Ni^3+^/Ni^2+^) increases from 1.924 in the pristine sample to 2.029 in the irradiated sample, indicating a higher concentration of the Ni^3+^ state present in the film. The decrease in the intensity of the core levels for Ni^2+^ and Ni^3+^ is more noticeable in the 5 minute sample, suggesting the could be due to ionization effects caused by microwave irradiation, which leads to the ejection of electrons from the Ni^2+^ and Ni^3+^ states. Additionally, defects induced by irradiation can alter the local environment around the nickel ions, reducing the observable intensity of these peaks. Furthermore, the binding energy difference (Δ*E*) between the Ni 2p_3/2_ and Ni 2p_1/2_ peaks is 17.4 eV for both the pristine and 5 minute samples. This confirms that nickel is present in its oxidized forms (Ni^2+^ and Ni^3+^).

Overall, the XPS investigation confirms that non-stoichiometric NiO has a higher proportion of Ni^3+^ in both the pristine and 5 minute samples. This analysis supports PL analysis, which also showed an increase in the Ni^3+^ state after irradiation. Therefore, it can be concluded that the film contains different states of nickel, with a higher concentration of the Ni^3+^ state in the nickel oxide film.

#### 3.3.3. O 1s core spectra

**Fig. 10:**
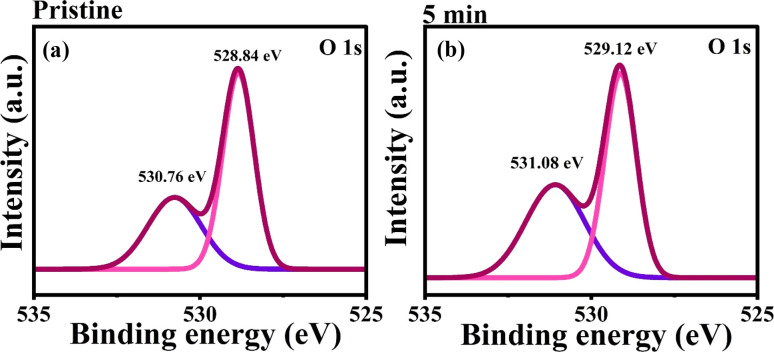
O 1s core level spectra of pristine and 5 min MW irradiated NiO nanostructure film.

The oxygen core level X-ray photoelectron spectroscopy (XPS) spectra of the oxygen 1s orbital have been analyzed by deconvolution using a Gaussian profile. The spectra were acquired over a binding energy range of 535 to 525 eV for both pristine and 5 min MW-irradiated NiO nanostructure thin films depicted in Fig. 10.

The deconvolution process revealed two predominant photoelectric emission bands of O 1s spectra. The pristine NiO thin films exhibit two distinct peaks located at 528.84 eV and 530.76 eV in the XPS spectra. The peak at the lower binding energy (528.84 eV) is attributed to lattice oxygen (O_L_) within the NiO matrix. This specific peak signifies the presence of oxygen atoms bonded with Ni^2+^ ions, forming the Ni–O octahedral coordination characteristic of the cubic rock salt structure.^42^ The presence of this peak indicates the stoichiometry of the NiO lattice,^43^ consistent with the XRD results.

The peak at the higher binding energy (530.76 eV) is associated with oxygen vacancies (O_V_) within the film.^44,45^ According to U. Kwon *et al.*,^46^ the presence of a peak around 531 eV can be attributed to a deficiency of Ni^3+^ ions and an excess of oxygen from NiO–OH. Similarly, research by Kotta *et al.*^47^ and R. S. Kate *et al.*^48^ supports this interpretation. M. Adak *et al.* also suggest that this peak corresponds to Ni^3+^ states in Ni_2_O_3_. The presence of oxygen vacancies has been confirmed by XPS analysis and is corroborated by results from PL spectra.

For the 5 min MW irradiated NiO film, the O 1s spectra exhibit peaks at 529.12 eV and 531.08 eV, respectively. These peaks indicate a shift towards higher binding energies compared to the pristine film. This shift, known as a chemical shift, reflects the alterations in the chemical states of the film induced by MW irradiation. The higher binding energy peaks suggest changes in the oxidation state and bonding environment of the oxygen atoms within the NiO lattice, highlighting the significant effect of MW irradiation on the film's chemical composition.

The area ratio of the oxygen 1s spectra for each peak was calculated, and it indicates that the percentage area of the lattice oxygen peak (O_L_) decreased from 61.7% to 54.1% upon MW irradiation. Conversely, the peak associated with oxygen vacancies increased from 38.3% to 45.9%. This change endorses the fact that MW irradiation enhanced the oxygen vacancy defects in the nanostructures, which is consistent with the PL analysis results.

### 3.4. FESEM and EDS analysis of MW irradiated NiO nanostructure films

**Fig. 11:**
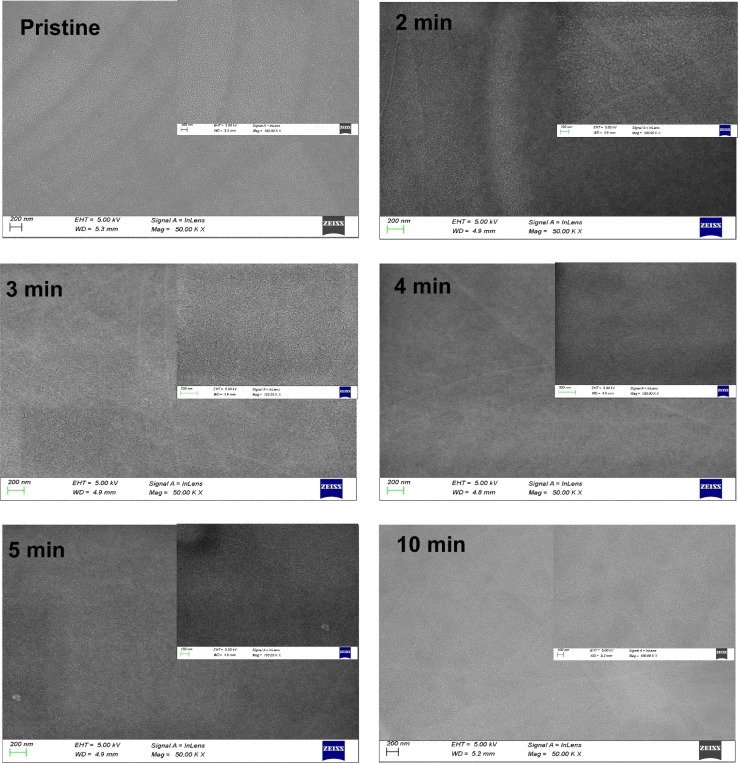
FESEM images of pristine and MW irradiated NiO nanostructure films.

The morphology of the nanostructure films was analyzed using FESEM, which examined the surface morphology of both pristine and MW-irradiated NiO nanostructure films, as shown in Fig. 11. All prepared films exhibited smooth, uniform, crack-free surfaces with nanosized grains and distinct boundaries. MW irradiation resulted in relatively smaller and smoother nanograins compared to the pristine films, indicating that MW irradiation affects the grain size. The reduction in grain size upon irradiation may be associated with increased lattice strain, which restricts lattice growth. This irradiation induces changes in the film's microstructure, leading to a more dispersed system.

EDS analysis was used to ascertain the elemental composition of NiO nanostructure films. In Table 3, EDS results for both pristine and MW-irradiated films indicate the exclusive occurrence of nickel (Ni) and oxygen (O) elements. With increased MW irradiation, the intensity of the O peak rises, indicating an increase in oxygen content in the films. Specifically, samples irradiated for 2 and 4 minutes show the highest oxygen peak intensity, likely due to a higher concentration of defect centers. This excess oxygen may result from the formation of Ni^3+^, which requires additional oxygen for charge compensation.^49^ Notably, XRD analysis did not detect any Ni_2_O_3_ phase in these films.^50^

**Table 3:** EDS results of MW irradiated NiO nanostructure films

MW irradiation	Ni wt%	O wt%	Ni at%	O at%
Pristine	67.76	32.24	36.42	63.58
2 min	52.88	47.12	23.42	76.58
3 min	67.07	32.93	35.69	64.31
4 min	56.97	43.03	26.51	73.49
5 min	60.19	39.81	29.18	70.82
10 min	68.92	31.08	37.67	62.33

### 3.5 . AFM analysis of MW irradiated NiO nanostructure films

**Fig. 12:**
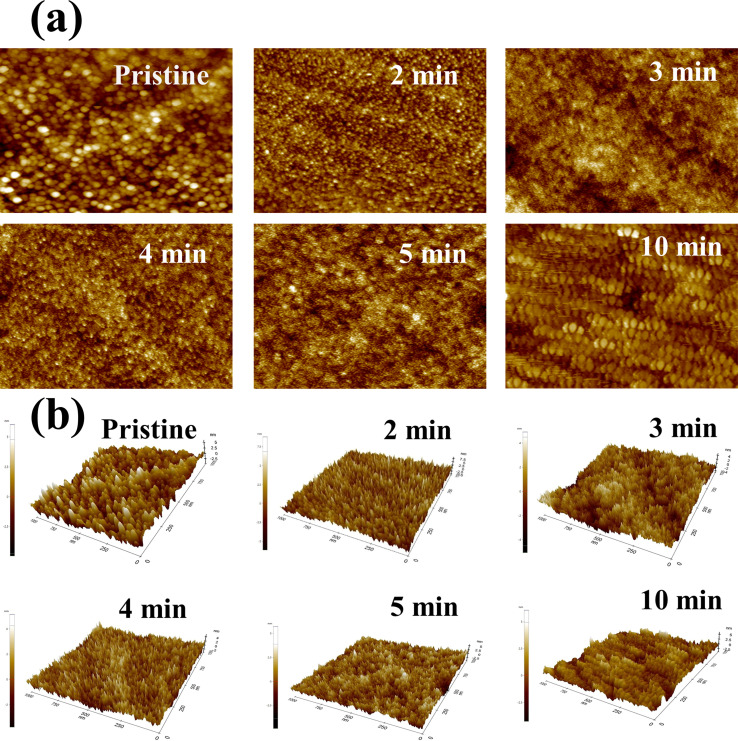
The (a) 2-D images and (b) 3-D images of pristine and MW irradiated NiO thin films.

The morphology of MW irradiated NiO thin films was examined using AFM techniques with tapping mode, and 2D and 3D images are presented in Fig. 12. The scan area of the film is 1 by 1 μm. The AFM analysis of NiO provides clear evidence of morphological changes in the film, including alterations in grain sizes, ordered arrangement, and film roughness after irradiation. The observable change in grain size is evident from pristine to 2 min of MW irradiation. In the AFM image of the 2 min sample, grain is more fragmented into smaller entities and found to be a more ordered way of arrangement than the pristine film. The morphology of the 3 min samples exhibits a cluster of grains, while the 4 min irradiation results in a distinct formation of well-defined grains. In the case of 5 minutes, a reduction in grain size is observed along with the formation of clustered grains. The 10 min irradiation sample showed an increase in the grain size, attributed to the attributed to the impact of irradiation. This implies that MW irradiation induces modifications in the grain distribution within the NiO film.

Variations in surface roughness were observed with increased MW irradiation. The surface roughness of the films is influenced by their crystallinity and texture; larger crystallite sizes generally result in smoother surfaces due to tighter packing of well-shaped grains. Table 4 shows the surface roughness values of the MW irradiated films. Values of roughness increased for the 2 minute irradiated sample, likely due to the presence of more dislocations and defects introduced by MW heating. However, with further irradiation, the roughness decreased, resulting in smoother surfaces due to reduced grain size and surface diffusion processes.^51^

**Table 4:** Surface roughness values of MW irradiated NiO thin films

MW irradiation	*R*_q_ (nm)
Pure NiO	1.091
2 min	1.645
3 min	1.176
4 min	1.058
5 min	1.083
10 min	1.076

### 3.6. UV-visible analysis of MW irradiated NiO nanostructure films

**Fig. 13:**
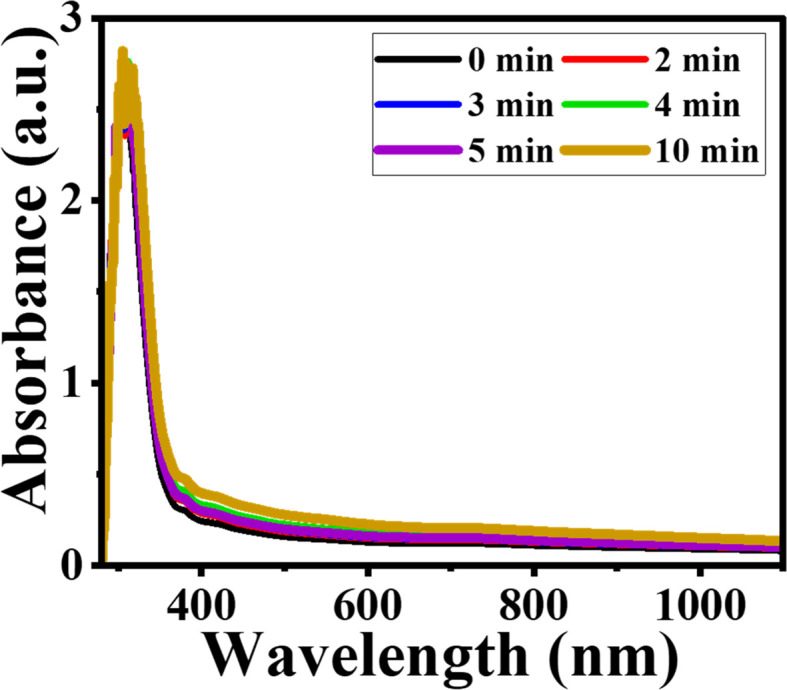
Absorption spectra of pristine and MW irradiated NiO nanostructure films.

The optical properties of the materials, including absorbance and energy bandgap, were assessed using a UV-visible spectrophotometer at ambient temperature. Fig. 13 shows the absorption spectra of both pristine and MW-irradiated NiO nanostructure films, with an inset providing a detailed view of the irradiation effects on NiO. High absorbance is noted in the UV range of 300–320 nm, whereas the visible and IR regions exhibit low absorbance. The high absorbance in the UV region is attributed to the bandgap absorption of NiO.^52^ V. Usha *et al.*^4^ suggest that a UV region broad peak is associated with π–π* inter-band transitions. S. Ghazal^53^ suggests that the occurrence of this high band is due to transition of the O 2p band to the Ni 3d state of the CB. The absorbance edge for NiO is around 348 nm, shifting to 356 nm after 10 minutes of irradiation, indicating a redshift associated with bandgap shrinkage. This redshift is associated with the increased presence of defect centers and heightened scattering losses from grain boundaries, ultimately leading to an enhancement in absorption. Additionally, absorbance increases upon irradiation, with the 10 minute sample showing high absorbance at 318 nm. This enhancement is likely due to the increased optical scattering loss of light from the grain boundaries. The increased absorption can also be seen in Fig. 1, likely caused by the enhancement of defects such as oxygen vacancies and increased Ni^3+^ ion concentration.^1^,^2^ These defect states act as centers, contributing to greater light absorption within the material, resulting in increased absorbance as MW irradiation increases. Similar results have been reported in the literature.^3^

**Fig. 14:**
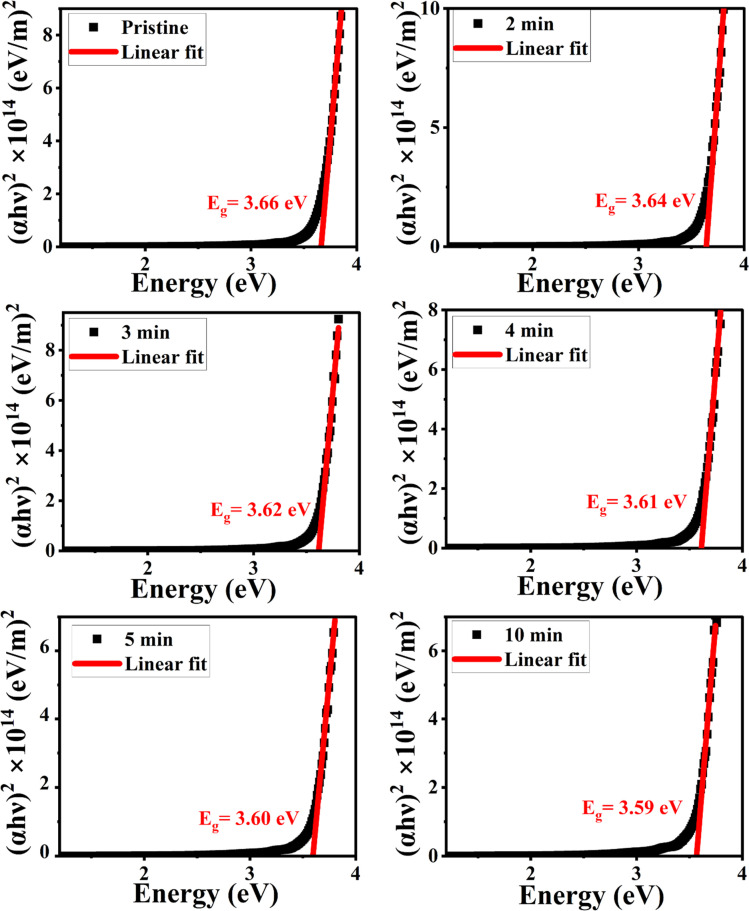
Energy bandgap of pristine and MW irradiated NiO nanostructure films.

According to structural investigations, irradiation enhances the defect densities in the lattice of the NiO nanostructure films. To confirm this change, we calculated the energy band gap of the pristine and MW-irradiated NiO nanostructure films, depicted in Fig. 14. The direct bandgap of the NiO films was determined utilizing Tauc's relation, given as:

8 (*αhν*)^2^ = *A*(*hν* − *E*_g_)

where *α* is the absorption coefficient of the film, *hν* is the photon energy and *A* is a proportionality constant. The linear section of the plots was extrapolated to estimate the film's *E*_g_ values and are provided in Table 5. The slight variations in the bandgap observed upon irradiation, which is associated with band filling. Irradiation increases the number of defect densities, leading to additional energy bands near the valence band (VB) and effectively decreasing the energy band gap. MW irradiation results in the formation of localized defect states, increased defect concentration, breakage of nickel and oxygen bonds, structural disorder, and enhanced strain in the film. This incorporation of localized defects causes a decrease in the energy bandgap.^17^ Furthermore, the reduction in bandgap is also associated with the compressive stress induced by MW heating in the lattice.^54^

**Table 5:** Energy gap values of MW irradiated NiO nanostructure film

MW irradiation	*E*_g_ (eV)
Pristine	3.66
2 min	3.64
3 min	3.62
4 min	3.61
5 min	3.60
10 min	3.59

### 3.7. Photoluminescence analysis of MW irradiated NiO nanostructure films

Photoluminescence (PL) spectroscopy provides critical insights into the crystal structure and defect densities within thin films. This technique is highly sensitive to defects, allowing for the detailed characterization of charge excitation and the electronic structure of the material. In our study, we investigated the PL spectra of pristine and microwave-irradiated NiO nanostructure films at room temperature, using an excitation wavelength of 300 nm.

The PL spectra exhibited a wide range of emissions extending from ultraviolet (UV) to visible regions, indicating the presence of various defects in the NiO nanostructure films. UV emissions were associated with band-to-band transitions, while the broad and intense visible emissions were attributed to deep-level intrinsic emissions, including nickel and oxygen interstitials and vacancies.

**Fig. 15:**
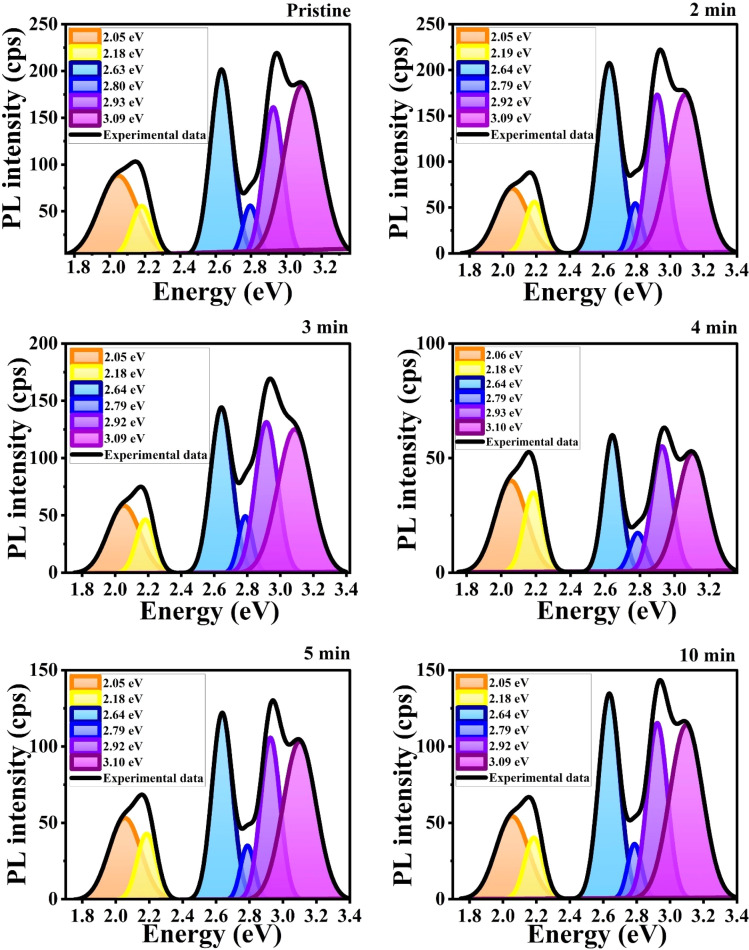
Gaussian deconvoluted PL emission spectra of MW irradiated NiO nanostructure films.

To precisely determine the luminescent emission centers corresponding to these defects, we performed a deconvolution of the PL spectra using a Gaussian distribution function. The results of this deconvolution are illustrated by the solid black line in Fig. 15. The fitted parameters obtained from this analysis are summarized in Table 6, providing detailed information on the specific defect states contributing to the observed PL emissions.

**Table 6:** Summary of PL peak fitted parameters of MW irradiated NiO nanostructure films

MW irradiation		UV	Violet	Blue I	Blue II	Yellow	Orange
Pristine	Intensity	189	161	56	201	56	88
	Area	47	22.6	5.7	31.5	7.7	23
	FWHM	0.24	0.13	0.10	0.15	0.13	0.25
2 min	Intensity	174	170	57	208	59	70
	Area	43.8	25.4	5.46	35.2	8.11	16.1
	FWHM	0.24	0.14	0.09	0.16	0.13	0.22
3 min	Intensity	125	132	49	143	46	57
	Area	33	22.4	5.26	23.8	6.56	14.5
	FWHM	0.244	0.16	0.10	0.15	0.13	0.13
4 min	Intensity	52	55	17	60	35	40
	Area	11.4	8.19	2.00	7.78	4.84	9.2
	FWHM	0.20	0.14	0.11	0.122	0.13	0.22
5 min	Intensity	103	106	35	122	43	53
	Area	25.7	15.7	3.75	18.8	6.2	13.02
	FWHM	0.23	0.014	0.10	0.014	0.013	0.22
10 min	Intensity	115	115	36	135	40	55
	Area	28.3	17.6	3.78	21.1	5.59	13.3
	FWHM	0.23	0.14	0.09	0.15	0.13	0.23

**Fig. 16:**
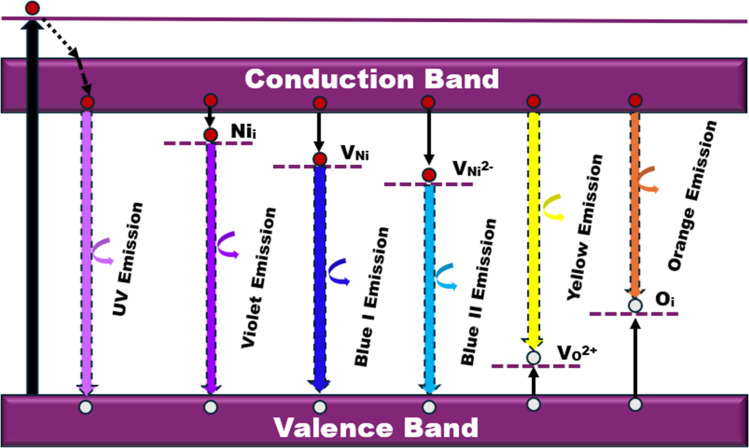
PL emission band diagram for different defect centers for NiO nanostructure films.

The PL spectra of pristine and MW-irradiated NiO nanostructures exhibit two prominent emission bands: one in the ultraviolet (UV) emission and another due to deep-level intrinsic emissions. The UV emission, corresponding to an energy of 3.09 eV, is accredited to near-band edge (NBE) excitonic recombination. The visible emission band, spanning from 2.93 to 2.05 eV, is associated with intrinsic defect levels caused by diverse structural defects, including oxygen vacancies and interstitial defects in the NiO films. Strong UV emissions arise from direct excitonic recombination near the band edge. F. Chandoul *et al.*^55^ suggest that the origin of this UV emission may be from oxygen vacancies in the NiO samples. The visible deep-level emissions are attributed to point defects, such as vacancies and interstitials within the nanostructured films.^56,57^ All visible transitions fall within the energy bandgap of the films, as determined from the Tauc plot, confirming that these defects are trapped within the material's bandgap. The deep-level emissions include violet emissions, broad blue emissions (blue I and blue II), as well as yellow and orange emissions across all prepared thin films. Fig. 16 illustrates the PL emissions resulting from various defect levels and the band edge transitions in NiO, providing a detailed band diagram representation.

The broad visible deep-level emission centers in MW irradiated NiO nanostructure films can be classified into two major categories. The first category includes emissions ranging from violet to wide blue, which are attributed to nickel-related defects within the films. The second category encompasses broad yellow to orange emissions, associated with oxygen-related defects.^58^ The energy emissions from 2.93 eV to 2.63 eV are linked to nickel defects, such as interstitials and vacancies in NiO materials. Mochizuki *et al.* suggest that the emission bands below 3 eV are attributed to nickel-related vacancy defects and occur due to charge transfer between Ni^2+^ and Ni^3+^.^59,60^ The emission at 2.93 eV corresponds to interstitial defects, attributed to the transition of electrons from nickel interstitial sites to holes in the valence band.^58^ Violet emissions arise due to the presence of nickel interstitial sites and surface defects, correlating with defect density. Blue I (2.80 eV) and blue II (2.63 eV) emission centers correspond to vacancy defects of nickel ions. The 2.80 eV emission is indicative of singly ionized nickel vacancies,^60,61^ while the 2.63 eV emission is attributed to doubly ionized nickel vacancies.^62^ Both transitions occur from nickel vacancy defect states to the valence band. The broad yellow to orange emissions detected in all nanostructured films, occurring at 2.18 to 2.06 eV, respectively, are attributed to defect-related emissions arising from oxygen vacancies.^63^ The 2.18 eV emission, appearing yellow, is attributed to the transition of electrons from the electron donor level to the acceptor level of oxygen vacancies. On the other hand, the orange emission at 2.06 eV is caused by oxygen interstitials.^64^ Also, the visible emissions occurred in the film due to the presence of defects at the grain boundaries, caused by the presence of nickel vacancies or the oxygen interstitials at grain boundaries.^60^

The effect of microwave irradiation on the position and intensity of defect peaks is illustrated in Fig. 15. While the peak positions remain stable with increasing MW irradiation, the intensity decreases in irradiated samples. A noticeable decrease in the variation of defect peak intensities was observed in the irradiated samples. The increase in irradiation creates defects such as vacancies, interstitials, and other surface imperfections, disrupting the regular lattice structure. These defects serve as non-radiative centers, leading to a decrease in PL intensity by dissipating energy as heat rather than light. Excessive defects further quench PL by forming non-radiative recombination centers.^65^ Moreover, the PL intensity indicates the dependency of defect density in the film, resulting in the weak recombination of photogenerated charge carriers.

There are no substantial deviations in the FWHM of the PL emission bands, but the area of the bands decreases with irradiation. The smallest band area was observed in samples irradiated for 4 and 5 minutes, indicating a higher number of defects and a reduction in luminescent centers. Additionally, the relative intensity between NBE emission and deep-level emission bands remains largely unchanged with irradiation.

After irradiation, a significant decrease in intensity was observed. Samples irradiated for 4 minutes exhibited a 75.76% reduction in UV emission intensity. This reduction is attributed to the microwave irradiation enhancing non-radiative defect levels in the lattice. XRD and Raman spectroscopy studies also support the incorporation of non-radiative defect centers upon irradiation. Ni interstitials were found to increase with irradiation, particularly in the 4 minute samples, while doubly ionized Ni vacancies decreased. The area ratio of nickel defects indicated an enhancement in nickel interstitials, with the highest levels observed in the 4 minute irradiated samples. Changes in the yellow-orange emission were also noted, with a decrease in oxygen interstitials and an increase in oxygen vacancies upon irradiation, consistent with XPS results. The calculated area ratios showed that the 5 minute film had the highest area ratio of 32% for oxygen vacancies, indicating a significant increase in oxygen vacancy defects. The area ratio quantity of the Ni and O defects were tabulated in Table 7. MW irradiation results in the decrease of PL intensity for NiO nanostructures, indicating that irradiation can effectively influence luminescence behavior. The quenching of PL emission with increasing irradiation dosage is due to the enhanced incorporation of non-radiative defect centers.

**Table 7:** Area ratio of Ni and O defects of MW irradiated NiO nanostructure films

MW irradiation	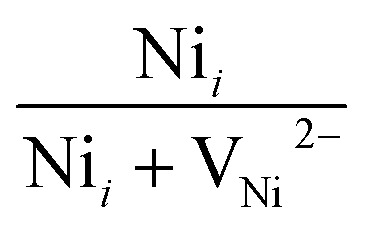	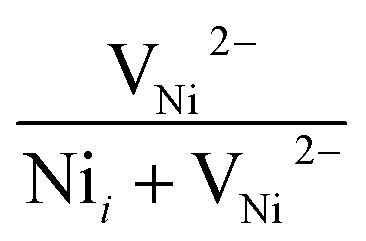	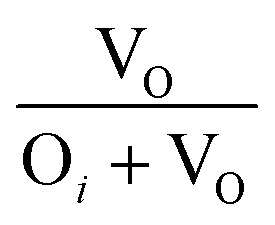	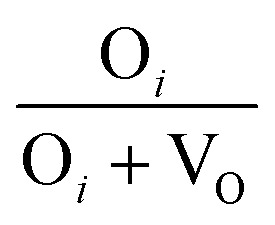
Pristine	0.4177	0.5822	0.2508	0.7492
2 min	0.4191	0.5808	0.3349	0.6650
3 min	0.4848	0.5151	0.3114	0.6885
4 min	0.5128	0.4871	0.3447	0.6552
5 min	0.4550	0.5449	0.3225	0.6774
10 min	0.4547	0.5452	0.2959	0.7040

### 3.8. Third-order nonlinear optical processes of MW irradiated NiO nanostructure films

#### 3.8.1. Open aperture Z scan technique

The nonlinear optical (NLO) properties of the pristine and microwave irradiated NiO nanostructure films were determined using a continuous wave (CW) laser with a wavelength of 632.8 nm. The Z-scan experimental method was employed to extract the nonlinear absorption (NLA) and nonlinear refraction (NLR) parameters using the open-aperture (OA) and closed-aperture (CA) approaches. The motivation behind this NLO experiment is twofold: to understand the effect of MW irradiation on NiO nanostructure films in both pulsed femtosecond laser and continuous wave laser regimes and to identify a suitable NLO material essential for current technological advancements. This section aims to elucidate the impact of continuous wave laser-induced nonlinearity in MW irradiated NiO films.

The theoretical understanding of NLO phenomena involves the interaction of high-intensity light, such as a laser, with an NLO material. The intensity of the light depends nonlinearly on the electric field. Consequently, the linear refraction and absorption become nonlinear and rely on the light's intensity as it passes through the material. This relationship is given by:

9 *α*(*I*) = *α*_0_ + *β*_eff_*I*

10 *n*(*I*) = *n*_0_ + *n*_2_*I*

where *α*_0_ and, *n*_0_, are the linear absorption coefficient and refractive index, respectively. *β*_eff_, and *n*_2_ are the nonlinear absorption coefficient and nonlinear refractive index, respectively. *I* is the intensity irradiance on the materials.

**Fig. 17:**
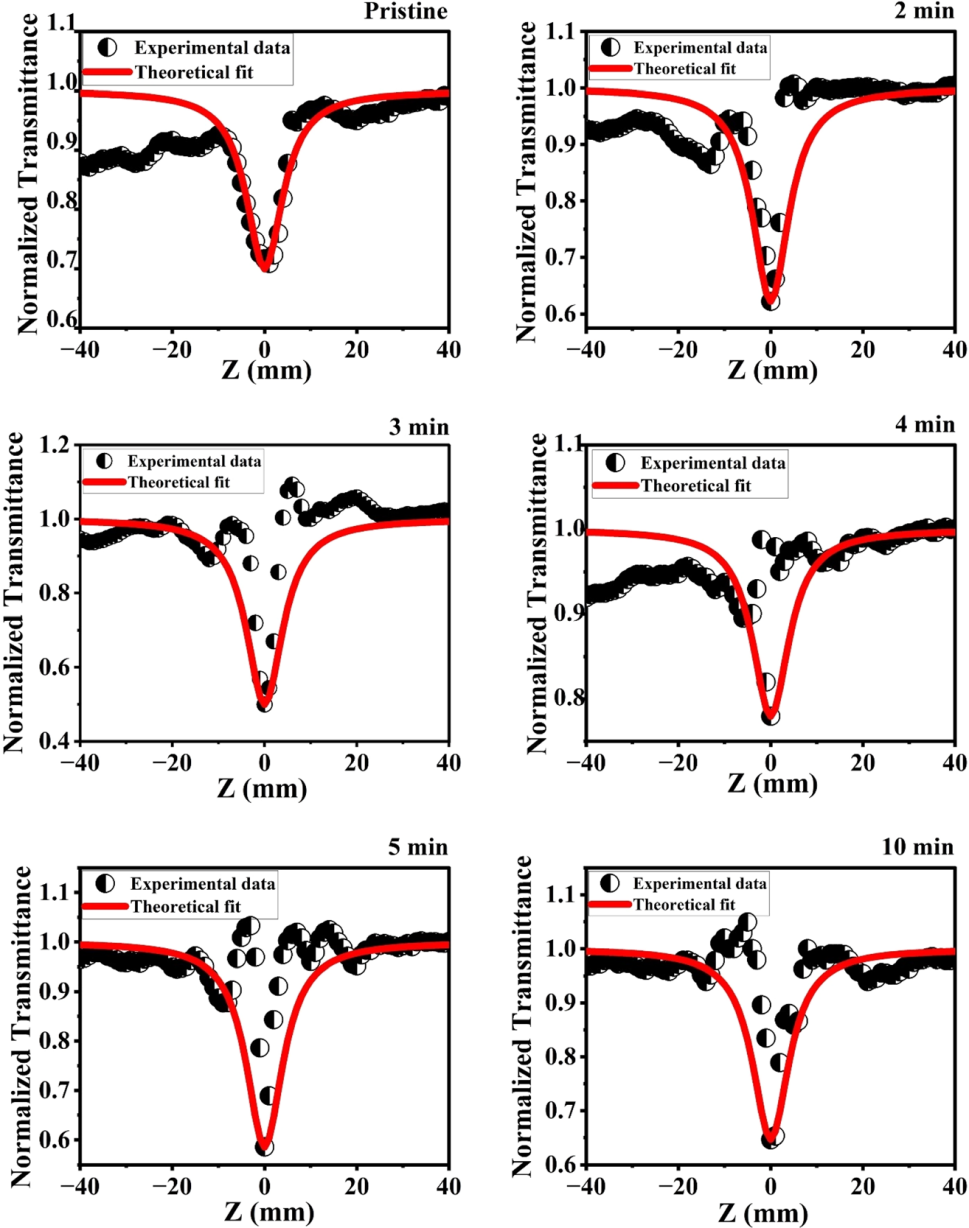
OA traces of the pristine and MW irradiated NiO nanostructure film.

The NLA coefficient *β*_eff_ of the material was determined using the OA Z-scan method, with an input intensity of 20 mW. The obtained results for the open-aperture Z-scan of the film, which correspond to the far-field normalized transmittance *T*(*Z*) as a function of the sample distance position from the beam focus, are shown in Fig. 17, represent the OA experimental data traces obtained with the Z-scan system. To obtain this graph, we used the general normalized transmittance equation for multiphoton absorption (MPA) in the OA Z-scan fit equation.11
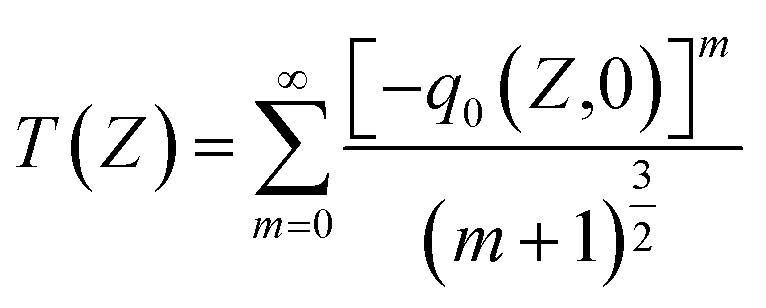
12
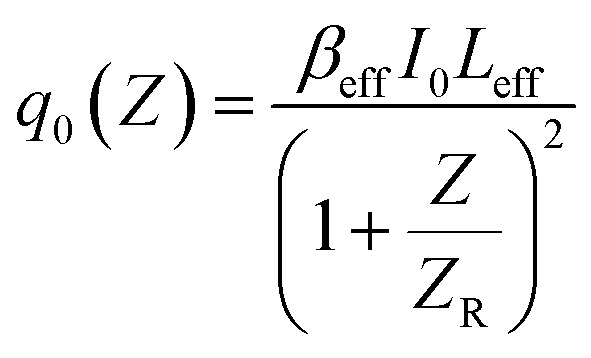
where *q*_0_(*Z*) is the free factor, *L*_eff_ the effective thickness of the material, *m*, is the number of photon absorptions.

The open-aperture graph exhibits a dip at the focus, indicating positive nonlinear absorption characteristic of reverse saturable absorption (RSA).^23^ Microwave irradiation enhances the RSA nature, with the valley significantly deepening MW irradiation compared to the pristine sample, indicating an increased nonlinear absorption mechanism.

The occurrence of RSA can be attributed to several mechanisms, including two-photon absorption (TPA), multiphoton absorption (MPA), free-carrier absorption (FCA), excited state absorption (ESA), transient absorption, or a combination of these nonlinear absorption processes.^66^ These behaviors are closely related to the bandgap, defect densities, and free carriers present in the films. The continuous laser irradiation energy of 1.96 eV on the nanostructured films excites electrons from the valence band to the conduction band through defect levels in the films, leading to thermal induced excited state absorption. The enhanced and induced defect levels in the films observed due to MW irradiation are associated with increased nonlinear absorption. In these nanostructures, electrons excited from the ground state to defect levels through photon absorption emit non-radiatively. These trapped electrons can further absorb additional photons, causing excitation to the conduction band, facilitated by ESA. Additionally, the laser irradiation energy of 1.96 eV is insufficient to excite electrons from the valence band to the conduction band through single-photon absorption. The bandgap of the material is ∼3.6 eV, and since the laser photon energy is greater than half but less than the bandgap energy, TPA is possible. The laser energy of 1.96 eV fulfills the TPA condition. FCA also plays a crucial role in the nonlinear absorption mechanisms. Microwave irradiation enhances the generation of free carriers within the material, thereby promoting FCA. Despite being a relatively weak nonlinear absorption process, FCA contributes significantly alongside TPA.^67^ Although TPA and the FCA-induced TPA are allowed, the high observed absorptive nonlinearity indicates that the RSA is primarily due to thermally induced ESA. Therefore, the RSA process primarily stems from ESA and FCA induced by TPA.

The nonlinear absorption results from the combined contributions of multiple processes, denoted by *β*_eff_. The presence and enhancement of defect levels in the films observed with MW irradiation, identified through structural and spectroscopic studies, provide additional levels in the bandgap, which enhance the ESA mechanism, leading to RSA in the film.

After MW irradiation of the NiO nanostructured film, the RSA behavior was found to be enhanced. Using the NLA data, we determined the nonlinear absorption coefficient, which is presented in Table 8. The increase in the NLA coefficient after irradiation underscores the significant impact of MW irradiation on the material. This enhancement is attributed to the increased defect levels, including structural imperfections, oxygen, and Ni^3+^ defects, which promote excited state absorption along with single, two, three, or multiphoton absorption, leading to NLA processes.^68^ Additionally, FCA further amplifies these mechanisms.^69^ The enhanced NLA after irradiation suggests that MW treatment effectively improves the material's nonlinearity.

**Table 8:** NLO parameters of the pristine and MW irradiated NiO nanostructured films

MW irradiation	*β*_eff_ × 10^−1^ (m W^−1^)	*γ* × 10^−7^ (m^2^ W^−1^)	*n*_2_ (esu) ×10^−1^	Real *χ*^3^ × 10^−2^ (esu)	Im *χ*^3^ × 10^−3^ (esu)	*χ* ^3^ × 10^−2^ (esu)
Pristine	3.57	0.83	4.94	1.64	3.56	1.68
2 min	4.85	1.31	8.44	3.24	6.04	3.30
3 min	6.47	1.33	8.71	3.45	8.42	3.55
4 min	2.93	0.94	6.71	3.20	5.05	3.24
5 min	5.39	1.76	11.8	4.95	7.65	5.01
10 min	4.76	1.11	8.22	4.22	9.15	4.32

These results indicate that the enhanced MW irradiation in NiO nanostructures leads to a greater NLA coefficient. It implies that the nonlinear absorption depends on the defect states induced by the irradiation, contributing to NLA and causing stronger NLA behavior in the MW-irradiated NiO nanostructured films. The results suggest that MW irradiation is suitable for enhancing the nonlinear optical absorption of NiO nanostructure films.

#### 3.8.2. Closed aperture (CA) Z scan technique

**Fig. 18:**
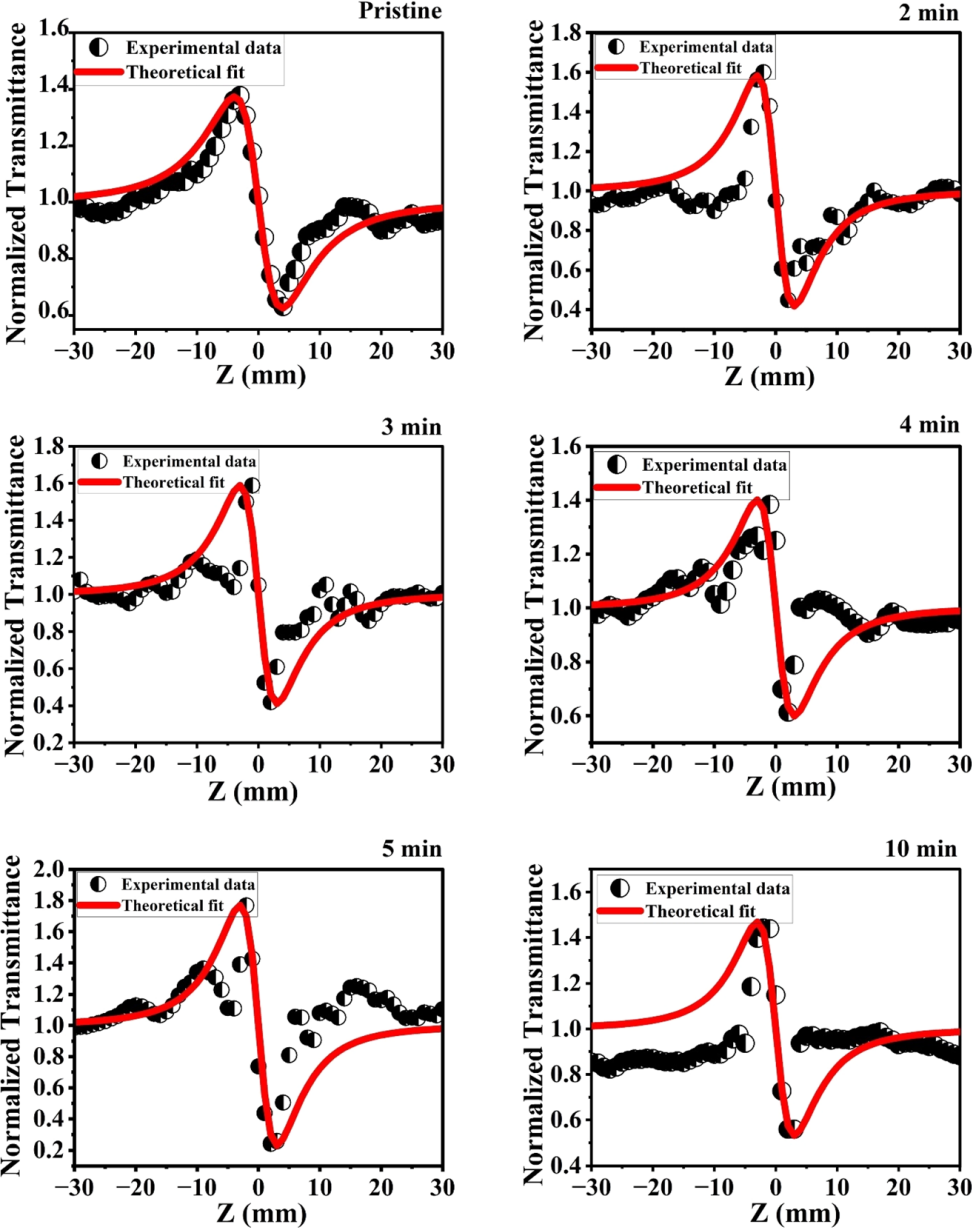
CA traces of the pristine and MW irradiated NiO nanostructure film.

The CA Z-scan technique was employed to determine the nonlinear refractive indices of pristine and MW-irradiated NiO nanostructure films. This method, proposed by Sheik-Bahae, helps calculate the sign and magnitude of the nonlinear refractive indices.^70^ To utilize the CA method, an aperture with a linear transmittance of 0.7 is placed in front of the detector. The sensitivity to nonlinear refraction is entirely due to the aperture and removing it would eliminate this effect. In the CA Z-scan method, normalized transmittance is plotted against the sample position (*Z*). In the obtained CA normalized transmittance data, the nonlinear absorption (NLA) effect is presented; it must need to be eliminated from the CA data. To eliminate this effect and acquire pure CA data, the CA data was divided by the OA data, which effectively ruled out the NLA effect. The resulting pure CA transmittance data, plotted against the sample position, is presented in Fig. 18.

The samples showed a pre-focal peak and a post-focal valley signature, indicating negative nonlinear refractive behavior, demonstrating a self-defocusing effect indicative of induced thermal lensing effects. The transmittance for the closed-aperture (CA) Z-scan is given below:13
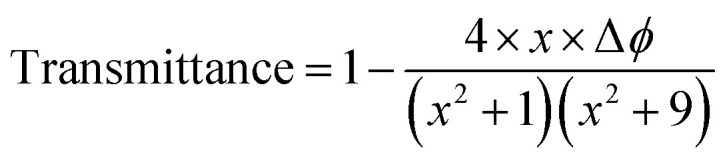
where 
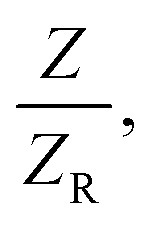
*Z* is the sample position and *Z*_R_ is the Rayleigh range. Δ*ϕ* is the on-axis nonlinear phase shift at focus. Normalized transmittance data is presented, with symbols representing the experimental data and solid lines depicting the theoretical transmittance fit to (5). Using the nonlinear refraction (NLR) plot of the material, we found the sign of the NLR indices to be negative. The nonlinear refractive index was calculated in both SI units and esu units, represented as *γ* (m^2^ W^−1^) and *n*_2_ (esu) respectively, and is given as follows:14
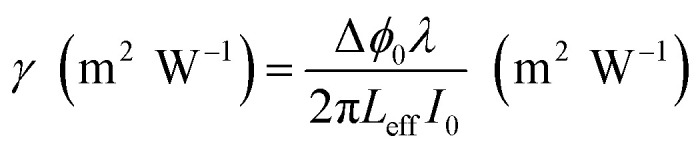
15
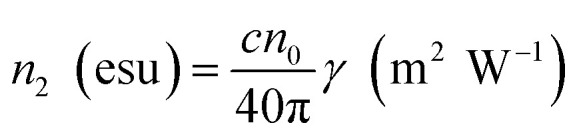
where *c*, *n*_0_, *L*_eff_, and *I*_0_ are speed light, linear refractive index, effective length, and intensity at focus, respectively. The values obtained of *n*_2_ obtained from fitting the NLR data are presented in Table 8.

The negative nonlinear refractive index in the samples is mainly caused by the thermal effects of the continuous wave laser. The CW laser irradiation on materials induces nonlocal thermal heating, which causes spatial variations in the refractive index of the material. These changes in the refractive index in the materials, which leads to materials to behave as a thermal lens due to the thermo-optic effect.^71^ The defect state levels in the MW irradiated NiO nanostructure films act as local thermal centers after absorbing incident photons and serve as centers for trapping excited electrons, enhancing non-radiative recombination. The non-uniform temperature distribution due to the irradiation causes variations in the refractive index of the MW irradiated material, which behaves like a defocusing lens, further leading to the enhancement in the negative nonlinear refractive index.

In the MW-irradiated NiO nanostructure film, peak-to-valley separation is greater than 1.7 times the *Z*_R_, strongly suggests that the nonlinearity predominantly arises from thermal effects.^72^ The nanomaterials exhibit absorption at the 633 nm wavelength, as seen in the absorption spectra. This absorption, combined with laser wavelength, leads to resonant nonlinearity, which further supports the presence of thermal nonlinearity in the material. Additionally, the CW laser typically enhances thermal nonlinearity through thermal lensing. Together, these factors confirm the dominance of thermal nonlinearity in this material.

Also, we employed the Z-scan technique to explore the effect of the glass substrate impacts the observed nonlinearity. Our results showed that the substrate did not exhibit any nonlinear optical effects, allowing us to eliminate the substrate's influence on our measurements.

In Fig. 17 and 18, we observe that the experimental Rayleigh length for MW-irradiated samples is narrower than the theoretical Rayleigh length. This is likely due to increased photon absorption processes, such as excited-state absorption (ESA), free-carrier absorption (FCA), and two-photon absorption (TPA). In this case, the nonlinearity is resonant, further amplifying these absorption processes. Additionally, the use of a CW laser contributes to thermo-optic nonlinearity, including thermal lensing, which plays a significant role in the increase in photon absorption.

The third-order susceptibility is a complex parameter that can be calculated using nonlinear optical parameters such as nonlinear absorption coefficient and nonlinear refractive index. The real part of the third-order susceptibility can be obtained from the nonlinear refractive indices, and it is given by:

16 Real *χ*^3^ (m^2^ V^−2^) = 2*n*_0_^2^*ε*_0_*cγ* (m^2^ W^−1^)

With the nonlinear absorption coefficient, the imaginary component of the third-order susceptibility can be computed and expressed as follows:17
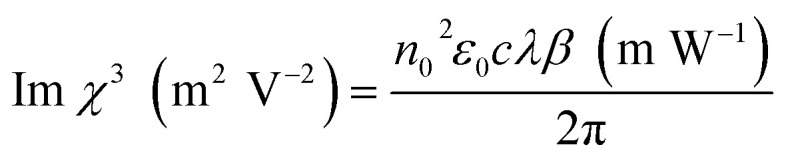


Then, the complex parameter third-order susceptibility can represent as,18
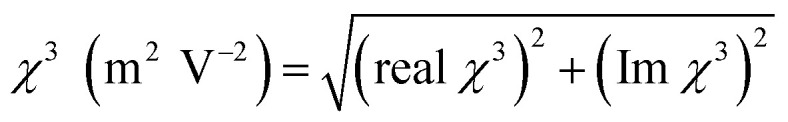


The below conversion relation helps in interchanging different units,19
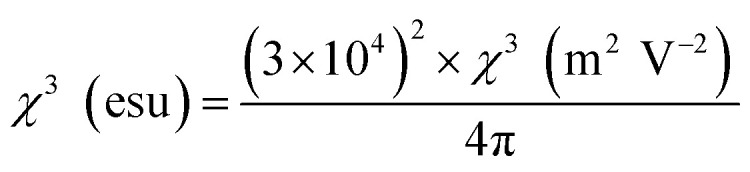


The nonlinear optical parameters, including the nonlinear absorption coefficient (*β*_eff_), nonlinear refractive index (*n*_2_), and third-order susceptibilities (*χ*^3^), were calculated and summarized in Table 8. Microwave irradiation led to an increase in these parameters, such as NLA, which was attributed to induced electronic transitions and the creation of intermediate states in the films. The variations in the nonlinear refractive index observed are associated with local heating effects induced by the laser light.

An increase in the *β*_eff_ observed with MW irradiation suggests that MW heating enhances the absorption nonlinearity of the NiO nanostructures. This enhancement is associated with the appearance of structural defects such as oxygen vacancies and Ni interstitials, which further lead to the enhancement of ESA, FCA, and TPA in the nanostructure. These enhancements result in the RSA mechanism being more pronounced, thereby increasing the NLA coefficient.

The higher values of the nonlinear refractive index and third-order susceptibility (*χ*^(3)^) observed with irradiation suggest the nanomaterial's potential for applications like optical limiting and optical storage. The third-order susceptibility (*χ*^(3)^) increased from 1.68 to 5.01 × 10^−2^ esu, with the highest value observed for the 5 minute irradiated sample. This improvement is attributed to the reduced surface roughness, which minimizes light scattering and enhances the optical properties. Additionally, the increased number of defect center formations in the 5 minute samples enhances the NLO mechanisms. These enhanced defect densities in nanostructures findings align with previous analyses such as XRD, Raman, XPS, PL, and UV analysis. A comprehensive Table 9 that summarizes the recently reported NLO parameters for various doped and undoped NiO at CW laser. As far as we know, the current data demonstrates the highest nonlinear parameters for NiO films under continuous wave prepared using physical and chemical deposition methods to date. This indicates that MW irradiation is an efficient method to enhance the NLO properties of NiO, rendering it a highly suitable material for numerous NLO applications.

**Table 9:** Reported nonlinear optical parameters of NiO and doped NiO at CW laser

Material	*β*_eff_ (m W^−1^)	*n*_2_ (m^2^/W)	*χ* ^3^ (esu)
Zn:NiO^73^	20.8 × 10^−2^ to 25.3 × 10^−2^	4.79 × 10^−8^ to 6.24 × 10^−8^	5.37 × 10^−3^ to 13.24 × 10^−3^
NiO:Cr^74^	3.19 × 10^−6^ to 3.98 × 10^−6^	2.24 × 10^−13^ to 2.85 × 10^−13^	3.74 × 10^−6^ to 4.32 × 10^−6^
NiO–Cu^75^	9.01 × 10^−2^ to 9.81 × 10^−2^	8.36 × 10^−9^ to 1.18 × 10^−9^	8.0 × 10^−8^ to 0.5 × 10^−8^
NiO:Al^76^	—	−8.67 × 10^−9^ to 11.67 × 10^−9^	—

#### 3.8.3. Third harmonic generation measurement

**Fig. 19:**
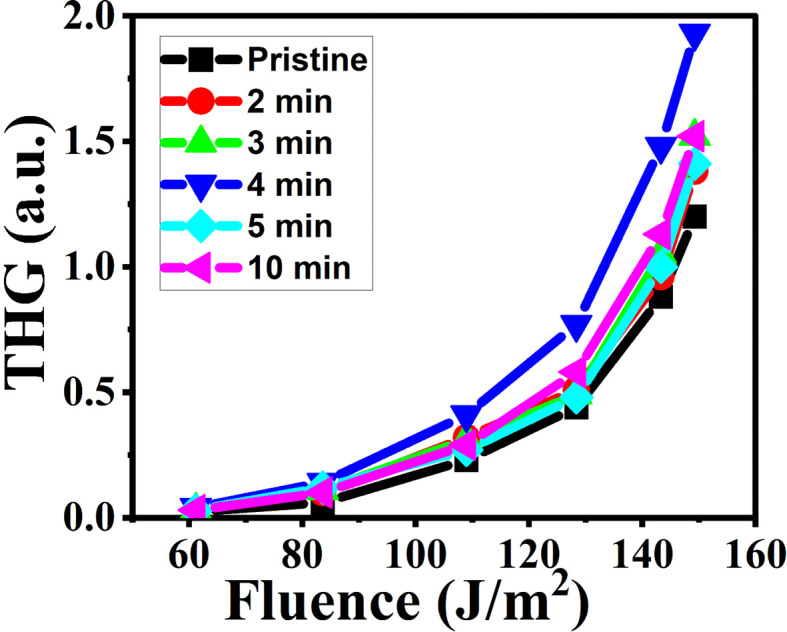
The illustration of THG signal with respect to the probing laser beam energy density of MW irradiated NiO nanostructure films.

In this discussion, we delve into the intriguing effects of third-harmonic generation (THG) signals concerning the energy density of probing laser beams and their impact on microwave irradiated samples. Fig. 19 serves as a visual representation, illustrating the THG signal's behavior concerning the variation in photoinduced probing laser energy density for both pristine and MW-irradiated NiO nanostructure films. This exploration sheds light on the complex interplay between laser energy, material properties, and THG responses, offering valuable insights into the underlying mechanisms of nonlinear optical phenomena in nanomaterials.

The THG signal varied with different probing laser beam energy densities from 60 to 150 J m^−2^. The plot reveals that there is no significant THG response for energy densities of the probing laser up to 60 J m^−2^. This suggests that below a certain threshold energy density, insufficient energy reaches the sample to generate a visible THG signal. However, as the energy density increases from 60 to 150 J m^−2^, there is a gradual enhancement in the THG signal. This enhancement is attributed to the interplay between photoexcitation and relaxation processes at localized trapping levels, a phenomenon also observed by other researchers^77^ with increasing power density.

For MW-irradiated NiO nanostructure films, the THG signal exhibits a non-monotonous trend. Initially, from pristine to 3 minute MW irradiation, the THG signal steadily increases. A significant enhancement is observed for the 4 minute irradiated films, followed by a decrease in THG for longer irradiation times. This behavior is explained by multiphoton excitation processes and optimized dipole moments, crystalline growth orientation, nanograin morphology, and optical properties.^78^ The enhanced THG signal for MW-irradiated films is attributed to increased optical absorption and excitonic effects. Enhanced optical absorption facilitates greater photon participation in the THG process, while excitonic effects contribute to photoexcitation and relaxation mechanisms. Overall, the interplay of these factors leads to the observed variations in the THG signal. The 4 minute MW-irradiated NiO sample shows a higher THG response than other samples, likely due to conditions favoring THG occurrence, increased photoexcitation and relaxation processes, and multiphoton absorption.^79^ This sample favors multiphoton excitation over third-harmonic processes, with interface trapping levels playing a significant role. Additionally, the 4 minute sample exhibits a low surface roughness value, contributing to the higher THG response.^80^ Furthermore, the laser-induced birefringence process substantially improves the THG signals through laser stimulation.^79^ These factors collectively contribute to the observed THG enhancement, attributed to improved photoexcitation and relaxation induced by the photo-induced pulsed laser. Non-regular increases in THG were observed by Upadhya *et al.*^81^ spray pyrolyzed Mg-doped ZnO films, where the enhancement in THG was attributed to the dipole moment of the excited states in the material. Albin *et al.*^82^ studied electron beam-irradiated GaZnO films under nanosecond and femtosecond laser regimes. They found significant results for both laser treatments, with enhancement in the femtosecond regime attributed to localized defects and laser-induced refractive index changes. In contrast, the enhancement in the nanosecond regime was primarily due to intra-energy gap states contributing to the NLO mechanisms. Essalah *et al.*^79^ reported a THG experiment with Nb_2_O_5_ and observed an increase in THG with rising Nb_2_O_5_ content. This enhancement was due to variations in atomic dipole moments, driven by mechanisms such as defect states, multiphoton processes, and additional photo polarization effects that alter the dipole moment of the atoms. Chattopadhyay *et al.*^83^ prepared Ce-doped ZnO *via* the coprecipitation method and found that the THG signal was enhanced when the dopant was in the interstitial position, while the THG signal decreased when the dopant substituted the ZnO nanoparticles. These findings align with the role of defect states in the observed variations in THG across different materials. Over 400 spots on the sample's surface were averaged in the results. After a few milliseconds, the photoinduced THG signal disappeared, demonstrating complete reversibility.^84^ Only when the photoinduced beam and the fundamental beam overlapped in both space and time was the THG signal recognized.

#### 3.8.4. Discussion on MW irradiation on third-order nonlinear optical processes on NiO nanostructure films

In summary, the third harmonic generation in microwave-irradiated NiO nanostructure films is significantly enhanced by femtosecond pulsed lasers. These ultrashort pulses interact with the film on extremely fast timescales, boosting the nonlinear optical response. The strong THG signal in the 4 minute irradiated sample is due to high irradiation-induced defects, which create energy levels within the bandgap. These defects facilitate transitions from localized states to the allowed band, enhancing the material's nonlinear optical properties. Conversely, the enhanced nonlinearity in MW-irradiated NiO under continuous wave laser irradiation is primarily thermal. The CW laser changes the refractive index through thermal excitation, amplifying the nonlinear response. The 5 minute irradiated sample shows strong third-order nonlinearity linked to exhibits lower surface roughness, resulting in a smoother surface as confirmed by AFM analyses.

Results from THG and Z-scan measurements reveal variations in nonlinearity origins due to the distinct influences of femtosecond pulsed and CW laser excitations. The femtosecond pulsed regime enhances the nonlinear optical response through ultrafast electronic excitations, making MW-irradiated NiO films suitable for frequency conversion in high-power laser sources. The CW laser regime's nonlinearity, driven by thermal effects, can be applied in optical limiting materials. These studies show that MW irradiation substantially enhances the nonlinear optical parameters of NiO nanostructure films, compared to recent data in the thermal nonlinear domain. This confirms MW irradiation as a direct strategy to control and manipulate the nonlinear optical properties of NiO films. The interplay of ultrafast electronic contributions from the pulsed regime and thermal effects from the CW regime suggests that MW-irradiated NiO nanostructures are promising materials for current nonlinear optical applications.

## 4. Conclusion

The pristine and MW-irradiated NiO nanostructure films with a face-centered cubic structure were synthesized using the chemical spray pyrolysis method. The XRD analysis reveals that crystallite size decreased upon MW irradiation, from 8.26 nm to 7.74 nm, due to the MW heating phenomenon. Raman spectra confirmed the characteristic NiO phonon modes for MW-irradiated NiO films, with a red shift towards higher wavenumbers and increasing intensity for the MW-irradiated samples, indicating increased strain and defect density within the nanostructures. The oxidation states of the NiO were quantified using XPS studies. FE-SEM images confirmed that the nanostructures have a smooth surface with nanograin-like structures. AFM studies showed that variations in surface roughness upon irradiation are due to changes in grain size and the ordered distribution of atoms within the lattice. The direct allowed band gap energies decreased from 3.66 eV to 3.59 eV for pristine and MW-irradiated NiO nanostructure films, respectively, confirming the narrowing of the band gap. PL quenching is observed in nanostructures with increased MW irradiation, associated with an enhanced quantity of nickel and oxygen defects within the lattice. The THG experiment showed an enhancement in the THG signal upon MW irradiation due to photoexcitation and relaxation processes occurring in the films, caused by increased defect densities in the lattice. All samples exhibit nonlinear reverse saturable absorption, as revealed by the open aperture Z-scan. Additionally, closed aperture measurements indicate the presence of negative nonlinear refraction, attributed to thermal lensing. The third-order nonlinear optical susceptibility showed an enhancement from 5.4 × 10^−3^ esu (pristine) to 4.76 × 10^−2^ esu (5 min) upon MW irradiation. The magnitude increased by two orders upon irradiation, implying that MW irradiation is a unique and novel method for enhancing the NLO mechanisms in these materials. Based on the experimental findings, it is evident that MW-irradiated NiO nanostructures demonstrate exceptional nonlinear optical characteristics, which highlights their potential for applications in optical detectors, offering superior protection against harmful laser radiation.

## Data availability

The data cannot be made publicly available upon publication as they are not available in a standard format that is sufficiently accessible by other researchers. The data that support the outcomes of this study will be shared upon reasonable request from the authors.

## Conflicts of interest

There are no conflicts to declare.
